# Manipulating the Assembly and Architecture of Fibrillar Silk

**DOI:** 10.1002/adma.202501096

**Published:** 2025-04-08

**Authors:** Chenyang Shi, Yuna Bae, Mingyi Zhang, James J. De Yoreo

**Affiliations:** ^1^ Physical Sciences Division Pacific Northwest National Laboratory Richland WA 99354 USA; ^2^ Department of Materials Science and Engineering University of Washington Seattle WA 98105 USA

**Keywords:** crystallization, in situ assembly, in situ biodegradation, nanofibril, silk

## Abstract

Silk is a unique and exceptionally strong biological material. However, no synthetic method has yet come close to replicating the properties of natural silk. This shortfall is attributed to an insufficient understanding of both silk nanofibril structure and the mechanism of formation. Here in situ atomic force microscopy (AFM) and photo‐induced force microscopy (PiFM) is utilized to investigate the formation process and define the basic structural paradigm of individual silk nanofibrils. By visualizing the multistage process of silk nanofibril formation, the importance of conformational transformations along the assembly pathway is revealed. Unfolded silk structures initially accumulate into amorphous clusters, which then evolve into crystal nuclei via conformational transformation into β‐crystallites. Nanofibril elongation then occurs through the attachment of silk molecules at a single end of the nanofibril tip; this is facilitated through the formation of a new amorphous cluster that then repeats the aforementioned conformational transformation. However, enzymatic digestion of the amorphous regions leads to direct, rapid elongation of β‐crystalline fibers. These findings imply that the energy landscape is characterized by shallow minima associated with intermediate states, which can be eliminated by introducing β‐crystallites, and motivate research into the directed modification of the silk assembly pathway to select for features beneficial to specific applications.

## Graphical abstract

In this study, advanced in situ AFM, PiFM, and Cryo‐EM are leveraged to realize in situ observations of nucleation, growth, and degradation of silk nanofibrils. A clear picture of the assembly and structural evolution of regenerated silk nanofibrils is defined and demonstrates a novel strategy for manipulating their structure and properties, which emerges from the understanding of their structure and assembly.

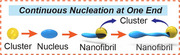

## 1 Introduction

Silk protein is renowned for its distinct mechanical properties, exceptional biocompatibility, and biodegradability.^[^ ^1^, ^2^, ^3^, ^4^ ^]^ The nanofibrils that form the basic unit within the intricate hierarchical structure of silk fibers (Figure S1a, Supporting Information) have long been suspected to play a pivotal role in elevating their mechanical performance^[^ ^5^, ^6^, ^7^ ^]^ as exemplified by the exceptional toughness of spider silk, which surpasses even that of steel and Kevlar.^[^ ^8^, ^9^, ^10^, ^11^ ^]^

Although nanofibrils are commonly found within bulk silk, a model for the structure, including the conformational state of the individual proteins, is not yet fully established. Most models propose that nanofibrils are semicrystalline and polymer‐like with highly oriented β‐sheet nanocrystals embedded in an amorphous matrix.^[^ ^12^, ^13^, ^14^ ^]^ Mechanically, the amorphous regions, which are comprised of unfolded silk, govern the elasticity,^[^ ^5^ ^]^ whereas the β‐sheet nanocrystals play essential roles in balancing the modulus, strength, and toughness.^[^ ^6^, ^15^, ^16^, ^17^ ^]^ However, unfolded structures exist in a thermodynamically metastable state and can spontaneously transition to the β‐form conformation under environmental regulation.^[^ ^18^, ^19^, ^20^, ^21^, ^22^ ^]^ This intricate molecular behavior involves a competition between the breaking of the original disordered hydrogen bonds and the formation of new, highly ordered bonds among the silk chains^[^ ^9^, ^17^, ^23^, ^24^ ^]^ (Figure S1b, Supporting Information). In other words, the unfolded silk molecules that constitute the matrix of the nanofibrils are in a dynamic state of stability. Thus, minor variations in microscopic conformation within the nanofibril can cause notable changes in the properties of the resulting macroscopic silk material.^[^ ^25^ ^]^ Therefore, deciphering the process by which the conformation of silk proteins within the nanofibrils evolves as the fibrils nucleate and grow is crucial for understanding and controlling the structure of the nanofibrils and thus the hierarchical architecture of the resulting bulk silk material in order to achieve high performance.

To understand the process of silk nanofibril formation, we combined in situ AFM and PiFM to both capture the initial stage of nanofibril formation and track the evolution of nanofibril structure and protein conformation. The results show that the assembly process follows a controllable, multistep pathway involving phase transitions in both the nucleation and elongation stages, which are characterized by unfolded silk molecules transforming into β‐form conformations. Moreover, we show that by modifying the energy landscape across which growth proceeds, we can alter the assembly pathway of silk molecules to construct a unique enzyme‐resistant silk nanofibril. The results provide a quantitative, mechanistic picture of silk nanofibril formation and define an innovative strategy for manipulating nanofibril structure to create advanced silk materials by tuning the energy landscape and the resulting pathway of silk protein self‐assembly.

## 2 Results

### 2.1 3D Morphology of Silk Nanofibrils

**Figure 1:**
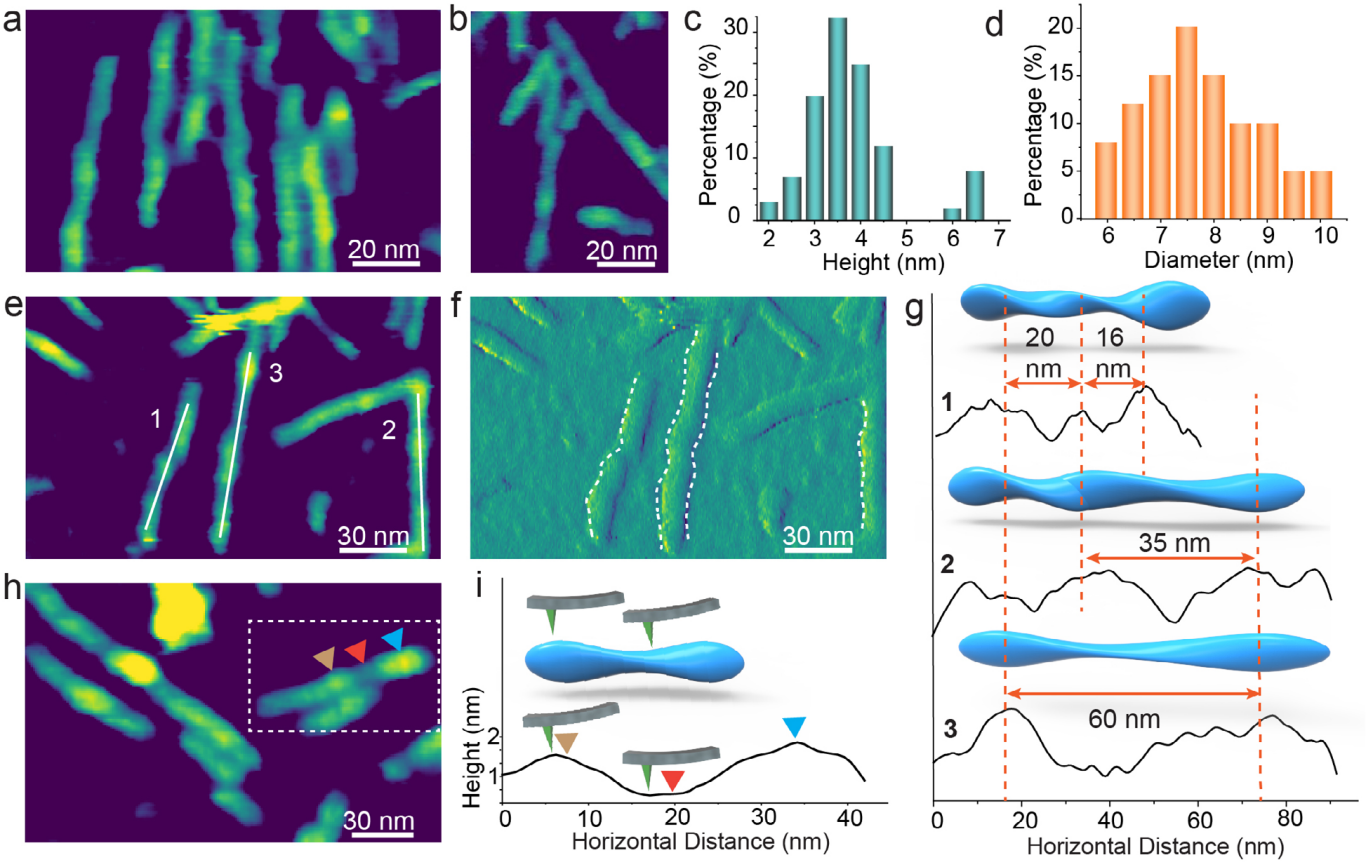
The structure of silk nanofibrils. a,b) In situ AFM height images of silk nanofibrils. c,d) Distribution of average nanofibril heights (c) and diameters (d) of silk nanofibrils formed during in situ AFM imaging. (sample size > 30). e,f) AFM height (e) and amplitude (f) images of silk nanofibrils. g) illustrations of helical pitch in the twisted geometry of the nanofibrils and the persistence lengths. h,i) AFM height image of the nanofibrils and a height profile along a cross‐section of a selected nanofibril, with corresponding local extrema indicated by colored triangles.

To assemble silk nanofibrils, we incubated silk protein solution at a concentration >20 µg mL^−1^ on graphite. AFM analyses of the nanofibrils’ morphological characteristics (**Figure** **1**a,b) showed that the average height was 3.8 ± 0.8 nm (Figure 1c), and the average width ranged from 10 to 14 nm (Figure 1d). When corrected for tip convolution, we estimate the true width to be 6–10 nm (see Experimental Section for details). Assuming the nanofibrils adopt an elliptical shape upon adsorption to the substrate, if these values of height and width are taken to be the diameters of an elliptical fiber, then the diameter of a circular fiber having the same cross‐sectional area would be 4.8–6.2 nm. CryoTEM images further support the values obtained from the AFM measurements giving a nanofibril diameter of ≈6 nm (Figure S2, Supporting Information).

Given that a typical fiber of natural silkworm silk is composed of many fibrils with diameters ranging from 20 to 100 nm, some studies suggest that these fibrillar structures are actually bundles of finer nanofibrils measuring ≈5 nm in diameter.^[^ ^26^ ^]^ Additionally, the height of individual nanofibrils chemically exfoliated from silk fibers has been reported to be 2–4 nm.^[^ ^27^ ^]^ The nanofibril we observed exhibited β‐sheet‐rich structures (Figure S3, Supporting Information), which is a defining feature of natural silk fibrils. Based on these findings, we conclude that the reconstituted nanofibrils in our study have dimensions and structures comparable to those extracted from natural silk fibers. These nanofibrils also exhibit a twisted structure with a heterogeneous axial pitch that varies between 16 and 60 nm (Figure 1g). Despite the heterogeneity of the pitch, the maximum height of the nanofibrils remains relatively consistent across fibrils, with only minor variations in the range of 0.5–1 nm (Figure 1h,i; Figure S4, Supporting Information). Thus, the AFM and CryoTEM data lead to a model for the morphology of the nanofibrils that consists of a twisted ribbon of constant diameter and variable pitch (Figure 1g,i).

### 2.2 Nucleation Process of Silk Nanofibrils

**Figure 2:**
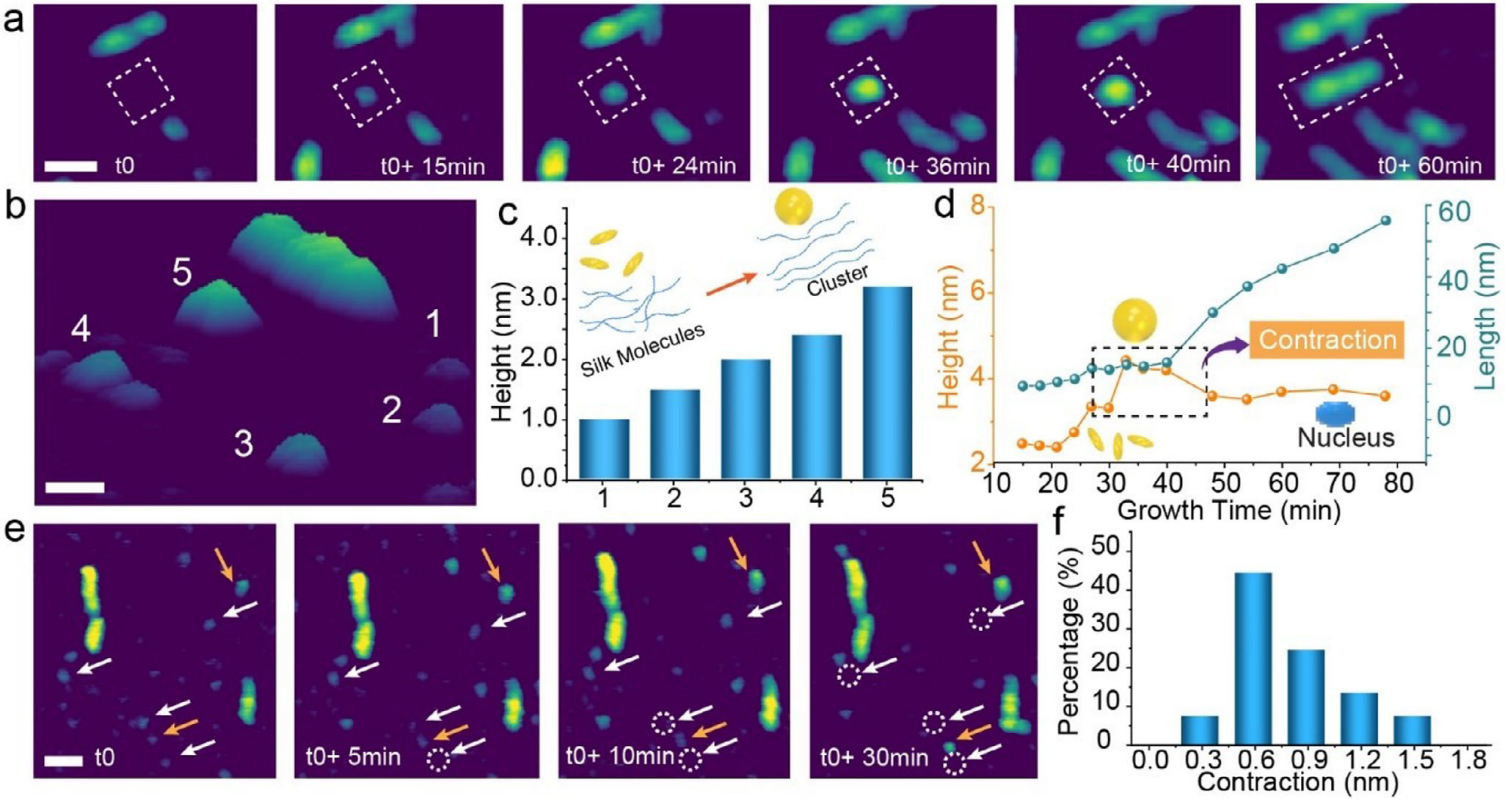
The process of silk nanofibril nucleation. a) In situ time series of AFM images showing silk nanofibril formation. Scale bar: 20 nm. b) Surface plot showing 3D topography of silk structures at various stages of formation. Scale bar: 10 nm. c) Heights of various objects from image (b) show that changes in height occur in increments with 0.5 nm during vertical growth. d) Height and length of a typical growing silk nanofibril vs time. The 3D models illustrate the shape change of the nanofibrils during the growth process. e) Time series of in situ AFM images showing the silk nanofibril nucleation process. Scale bar: 20 nm. The white arrow indicates the particle that may disappear, while the orange arrow marks the nuclei. The dashed circle highlights the disappearance of the cluster. f) Percentage of nanofibril ends exhibiting various values of vertical contraction during nucleation (sample size > 30).

Following the characterization of the structure, we then studied the mechanism of nanofibril formation using time‐resolved in situ AFM. When incubating silk protein solution (50 µg mL^−1^) over 10 mins, we first observe the appearance of small particles with a height of 1 nm or less, which gradually grow in height until they reach 3–4 nm (**Figure** **2**a). Moreover, the vertical growth occurs in regular increments of ≈0.5 nm (Figure 2b,c), which is close to the dimension of an individual silk molecule,^[^ ^27^, ^28^ ^]^ indicating an orderly vertical addition of individual silk molecules during the initial stage of nanofibril formation. Once these particles reach ≈4 nm in height, they contract to ≈3 nm, which is the diameter measured for mature nanofibrils (Figure 1c), and begin to undergo longitudinal growth without any further change in height (Figure 2d; Figure S5, Supporting Information). Additionally, we found that some of these particles grow while others disappear during the assembly process (Figure 2e). Thus these initial particles are akin to the fluctuating sub‐critical clusters in nucleation theory and must reach a critical size before growth can spontaneously occur.^[^ ^29^, ^30^ ^]^ However, the contraction in height suggests that a key step that leads to elongation is a conformational change that leads to a more compact structure, indicating a two‐step nucleation process in which an initial phase that is only stable at microscopic dimensions converts to a stable configuration once it exceeds a size at which there is a crossover in phase stability.^[^ ^31^, ^32^, ^33^, ^34^ ^]^ Nanofibril elongation begins immediately upon the cluster contraction (Figure 2d), which typically averages 0.5 nm but is as large as 1.5 nm (Figure 2f).

### 2.3 Elongation Process of Silk Nanofibrils

**Figure 3:**
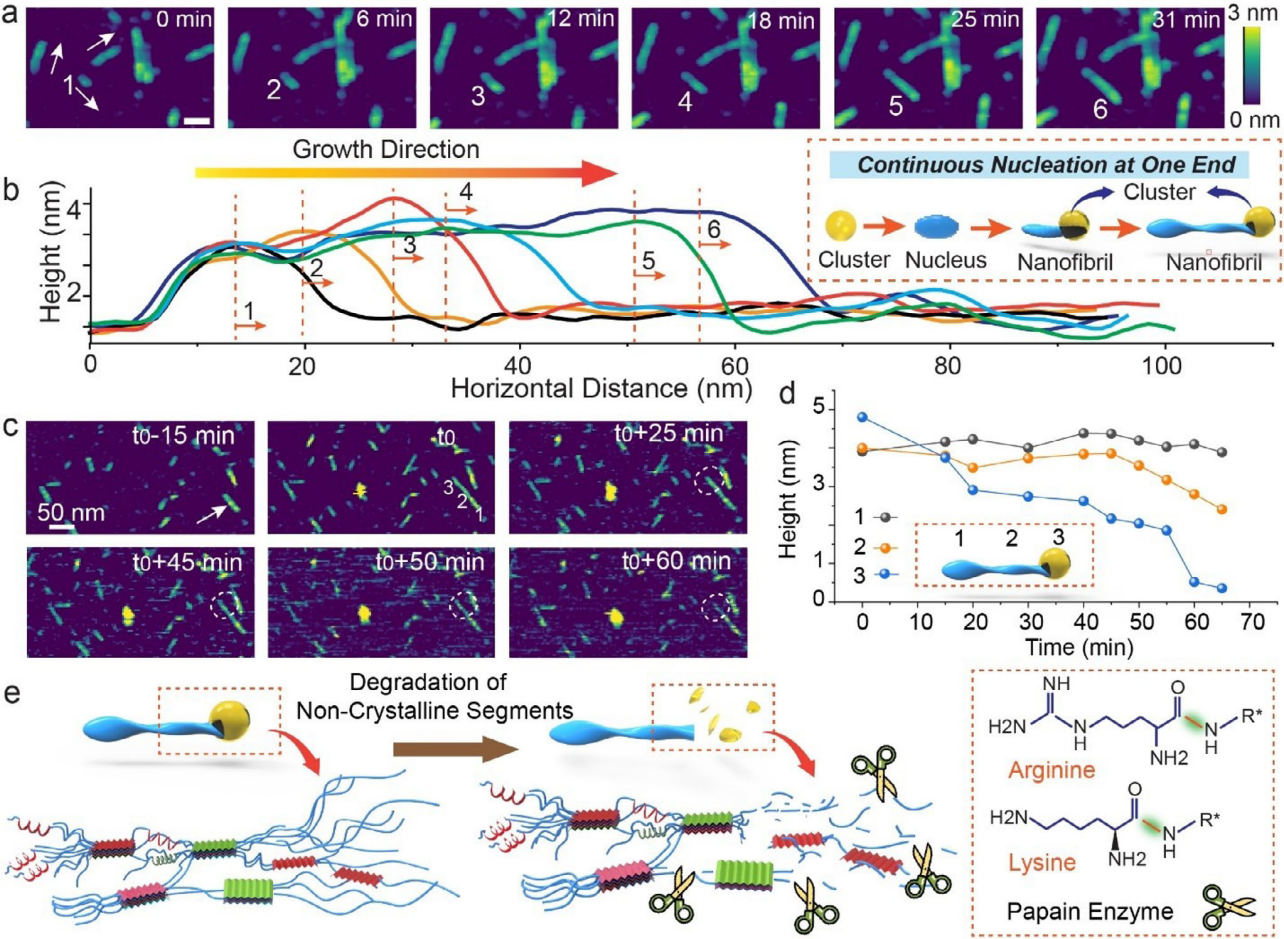
The process of silk nanofibril elongation. a) Time series of in situ AFM images showing silk nanofibril growth. Scale bar: 20 nm. b) The corresponding height profile of silk nanofibrils vs growth time. The numbers 1–6 correspond to the labels of the silk nanofibril in panel (a) imaged at consecutive growth times. The 3D models in the dashed box illustrate the continuous monodirectional growth of the nanofibers. c) AFM images depicting the enzymatic degradation of a silk nanofibril. d) Variations in height vs degradation time for spots labeled 1, 2, and 3 in Figure 3c. e) Illustration of the selective degradation mechanism for the papain enzyme.

Once the silk nanofibril nucleus converts to the compact structure, it spontaneously grows longitudinally by subsequent attachment of silk molecules to the tip (**Figure** **3**a), though growth sites can also occur occasionally along the body of the nanofibril, inducing branch growth (Figure S6, Supporting Information). Moreover, as with the initial nucleus, the height of the growing tip is greater than the body of the nanofibril by 0.5–1.5 nm and contracts as the nanofibril elongates further (Figure 3b). Finally, in almost all cases, growth occurs at just one end and the growth direction remains fixed once growth begins, with no elongation occurring in the opposite direction. This observation implies that the nanofibril has a polarity that allows for growth at only one end, as is reported for several natural protein fibers.^[^ ^35^ ^]^

The conformational transition of the nucleus, as well as the growing tip, is reminiscent of other crystallizing and self‐assembling systems in which the initially formed particles are amorphous and become more compact as the system develops order.^[^ ^34^, ^36^, ^37^ ^]^ In order to investigate the conformational state of the nanofibril tip, including the possibility that it is amorphous, we used time‐resolved in situ AFM data to observe enzymatic degradation of silk nanofibrils (Figure 3c,d). Enzymatic degradation of silk materials leads to their breakdown into smaller polypeptides and, eventually, to amino acids.^[^ ^38^, ^39^, ^40^ ^]^ More importantly, enzymatic degradability is strongly sensitive to protein conformation.^[^ ^39^ ^]^ Specifically, the cleavage sites of proteolytic enzymes in the silk amino acid sequence are different for distinct conformation states.^[^ ^39^ ^]^ For example, papain can hydrolyze peptide bonds formed by the carboxyl groups of lysine and arginine and adjacent amino acid residues typically present in the non‐β‐sheet crystalline segments of the silk molecule,^[^ ^39^ ^]^ thus selectively degrading the non‐crystalline regions, whereas protease XIV has the capability to degrade all types of silk molecule conformation regardless of crystallinity.^[^ ^39^, ^41^, ^42^ ^]^

Upon injection of fresh papain solution, we find that silk nanofibrils stop growing and the tips degrade first (Figure 3c–e). In contrast, when silk nanofibrils are treated with protease XIV, they exhibit a complete breakdown of the entire silk nanofibril both at the tip and throughout the body of the nanofibril (Figure S7, Supporting Information). In addition, Young's modulus at the nanofibril tip is relatively lower than that of the body, indicating a softer and less structured region at the tip (Figure S8, Supporting Information). Thus, we conclude that the tip is dominantly comprised of unfolded silk molecules. By extension, we assume that the initial particles that have not yet reached critical size and undergone contraction are also dominantly comprised of unfolded silk molecules.

**Figure 4:**
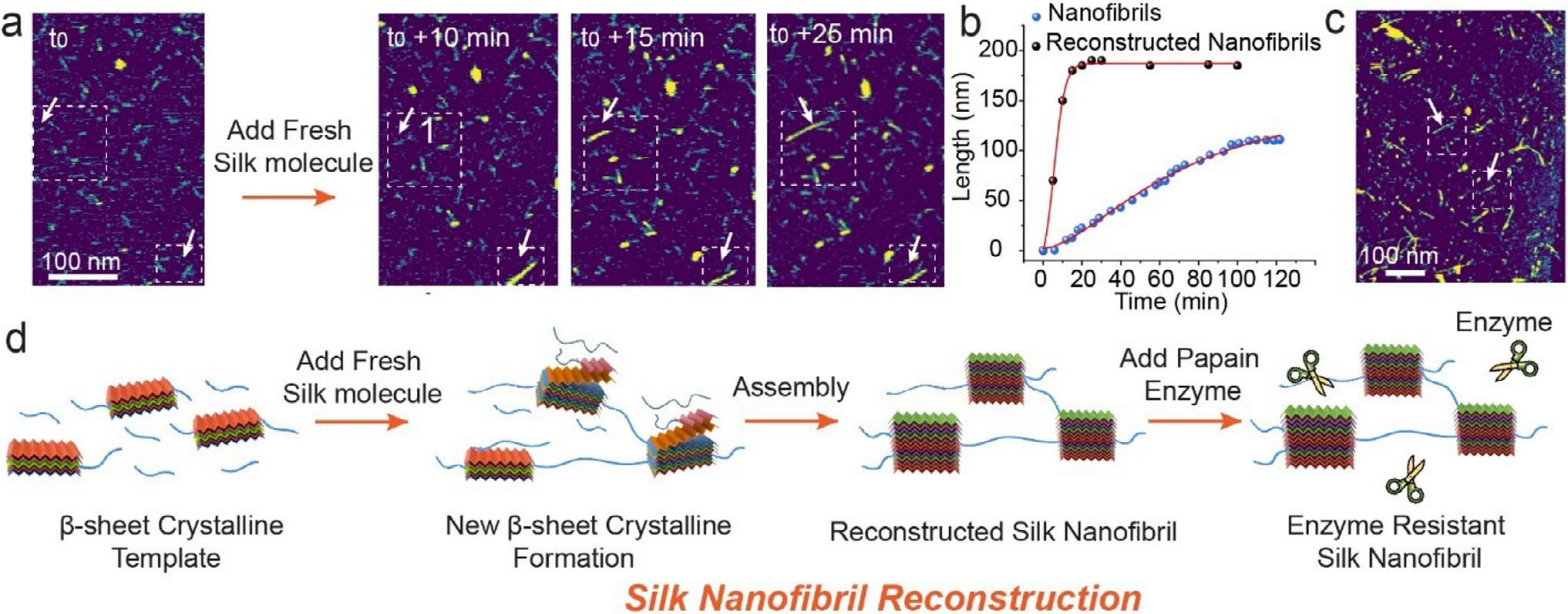
Reconstruction of silk nanofibrils using enzyme‐treated β‐sheet crystals as templates. a) AFM images of reconstructed silk nanofibrils. The white arrows point to the target nanofibrils. b) The growth length of silk nanofibrils vs growth time. The reconstructed silk nanofibrils show a remarkably rapid growth rate compared to normal silk nanofibrils. c) AFM images of reconstructed silk nanofibrils after treatment by fresh papain enzyme. The white arrows point to the same target nanofibrils with panel (a). d) The scheme of reconstructing silk nanofibrils through modifying the assembly pathway.

We also observed that, after enzymatic degradation of the non‐β‐sheet crystalline segments, the remaining silk particles can serve as seeds that template the attachment of fresh silk molecules (**Figure** **4**a), leading to the rapid growth of larger nanofibrils, which are 2 nm taller than typical silk (Figure S9, Supporting Information), elongate at higher speed (Figure 4b), and exhibit resistance to enzymatic degradation (Figure 4c,d).

### 2.4 Conformation of Silk Nanofibrils

To investigate the structural makeup of the nanofibrils, we employed PiFM, a highly sensitive and spatially resolved nano‐spectroscopic scanning probe method that can be used to determine local protein conformation.^[^ ^43^ ^]^ This is accomplished by correlating morphology obtained from AFM with FTIR spectral signatures of secondary structure. In general, the conformational changes in protein structure are characterized by shifts in the vibrational bands of the amide I peak (1600–1700 cm^−1^), which correspond mainly to C═O stretching vibrations.^[^ ^20^ ^]^

**Figure 5:**
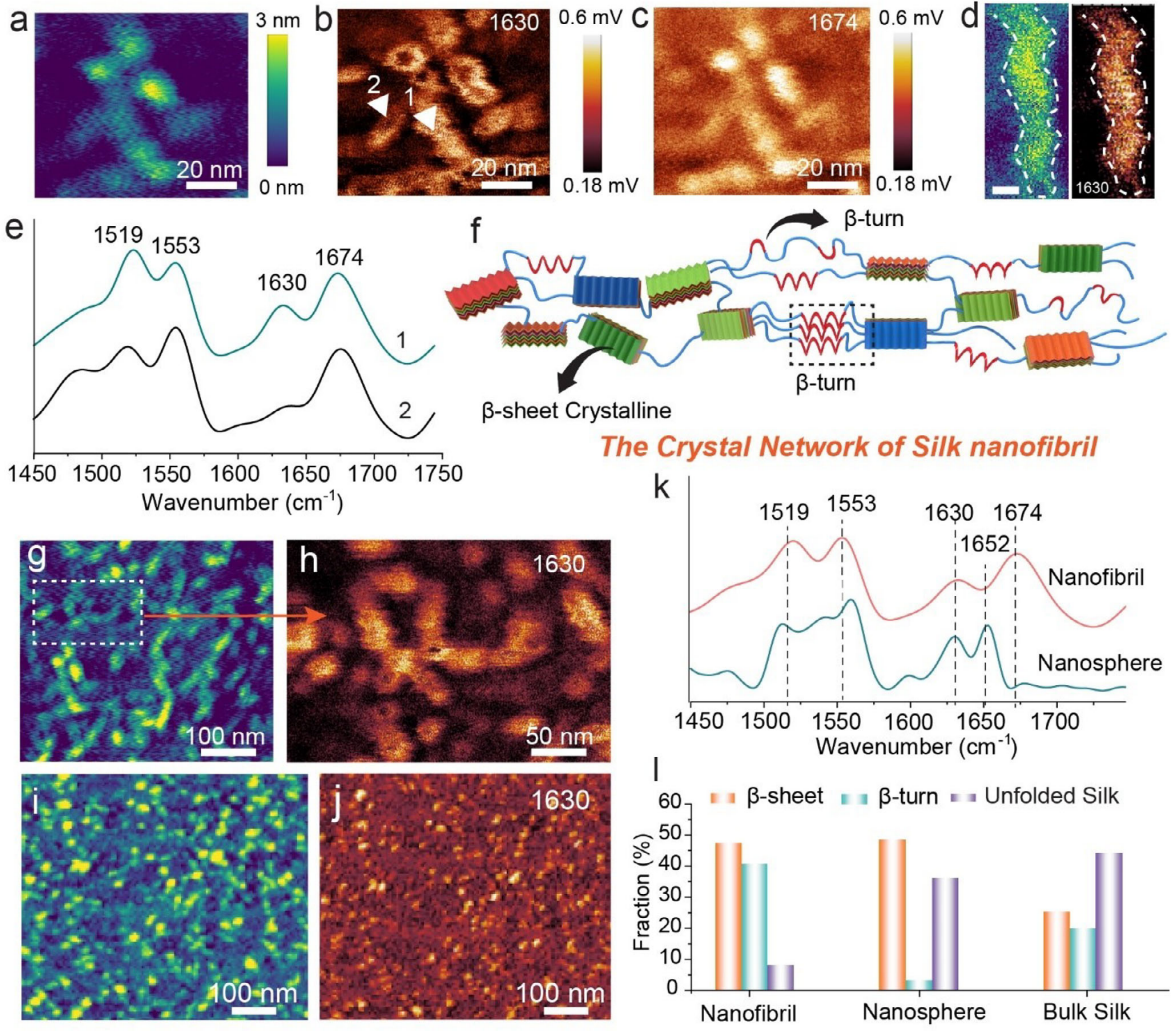
Analysis of the conformational structure of silk. a–c) AFM image of silk nanofibrils (a), and corresponding PiFM images at 1630 cm^−1^ (b) and at 1674 cm^−1^ (c). d) AFM image and corresponding PiFM image of an individual silk nanofibril. e) PiFM spectra of the silk nanofibrils in (b). f) Proposed crystal network of silk nanofibril. g) AFM image of silk nanofibrils. h) PiFM image from the white dashed box in (g). i,j) AFM image (i), and PiFM image (j) of silk nanospheres at 1630 cm^−1^. k) PiFM spectra of silk nanofibrils and nanospheres. l) Distribution of molecular conformations in various formats of silk.

As depicted in **Figure** **5**a–c, the PiFM images reveal the expected intense absorption at 1630 cm^−1^ (Figure 5b) and 1674 cm^−1^ (Figure 5c) associated with β‐sheet and β‐turn conformations,^[^ ^20^ ^]^ respectively. The high resolution of our PiFM results both in terms of spatial and spectral resolution allows us to discern the conformational variability of individual silk nanofibrils at the nanoscale, revealing subtle differences in conformation between distinct nanofibrils (spot 1, spot 2 in Figure 5b, spectrum in Figure 5e). Additionally, the spectra show that the conformation is largely homogeneous within an individual nanofibril, despite the heterogeneous pitch, as the intensity of the PiFM signal at each point along the nanofibril shows no significant variations (Figure S10, Supporting Information), suggesting the conformational transition is homogeneous during nanofibril elongation.

To analyze the conformational state of the proteins, an average infrared spectrum representing the protein signals was decomposed into a series of small bands representing the possible conformations, such as β‐sheet and random coil (Figure S11, Supporting Information).^[^ ^21^, ^44^ ^]^ The analysis indicates that the nanofibrils are primarily composed of β‐form structures, including β‐sheet and β‐turn (Figure 5f), in which silk molecules are known to pack together via hydrogen bonding, augmenting their thermodynamic stability.^[^ ^6^ ^]^ In contrast, the content of β‐sheet structures in silk materials that are not comprised of nanofibrils, such as nanospheres, is very low, while the content of random regions is high (Figure 5g–j). These analyses indicate that, during the nanofibril growth process, the silk molecules generate more intramolecular hydrogen bonds through the bending and folding of random structures into β‐turn and β‐sheet structures, thus reducing their free energy and achieving higher stability.

### 2.5 Nanomechanical Properties of Silk Nanofibrils

**Figure 6:**
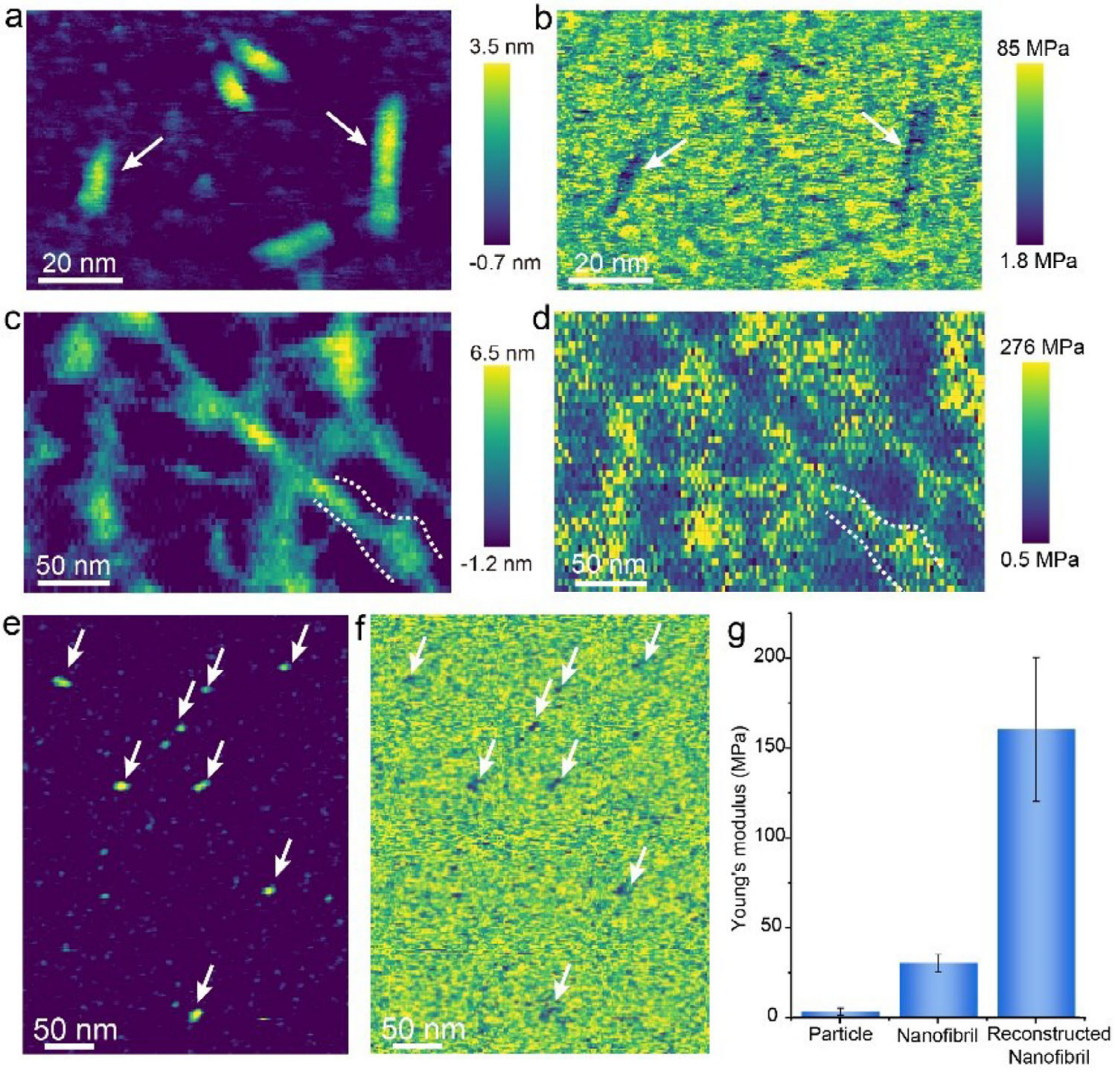
AFM‐based nanomechanical measurements in liquid. a) AFM image of nanofibrils and b) corresponding map of Young's modulus giving a value of 30 ± 5 MPa. c) AFM image of reconstructed nanofibril and d) corresponding map of Young's modulus giving a value of 160 ± 40 MPa. e) AFM image of silk nanoparticles and f) corresponding map of Young's modulus giving a value of 4 ± 2 MPa. g) Histogram of Young's moduli for the three different silk structures.

AFM‐based nanomechanical analysis reveals that silk nanofibrils exhibit a relatively low Young's modulus of ≈30 MPa in aqueous solution (**Figure** **6**a,b), whereas the reconstructed nanofibrils exhibit a significantly higher Young's modulus of ≈160 MPa (Figure 6c,d), which represents a fivefold increase over that of unmodified nanofibrils (Figure 6g). This shows that the elimination of the amorphous domain through reconstruction enhances nanofibril stiffness, likely due to improved β‐sheet organization and content.^[^ ^6^ ^]^ Since robust mechanical properties in biological materials are closely linked to β‐sheet composition,^[^ ^13^, ^24^, ^45^ ^]^ these results support the structural findings regarding enhanced β‐sheet optimization in reconstructed nanofibrils.

Figure 6e–g shows that some silk nanoparticles, which represent the initial phase of silk nanofibril assembly, exhibit a significantly lower Young's modulus than the nanofibrils. This finding is potentially linked to differences in molecular conformation between the nuclei of silk nanofibrils and the mature nanofibrils.

To enable direct comparison with reported nanomechanical properties measured in air, we also applied PeakForce QNM (Quantitative NanoMechanics) to nanofibrils in the air (Figure S12, Supporting Information). In the air, dried regular nanofibrils exhibited a large Young's modulus of ≈25 GPa, which is slightly higher than the best‐reported values.^[^ ^20^ ^]^ Surprisingly, the reconstructed nanofibrils achieved an average Young's modulus of 70 GPa—nearly three times higher than regular nanofibrils—further demonstrating the enhanced stiffness achieved through the reconstruction method.

## 3 Discussion

**Figure 7:**
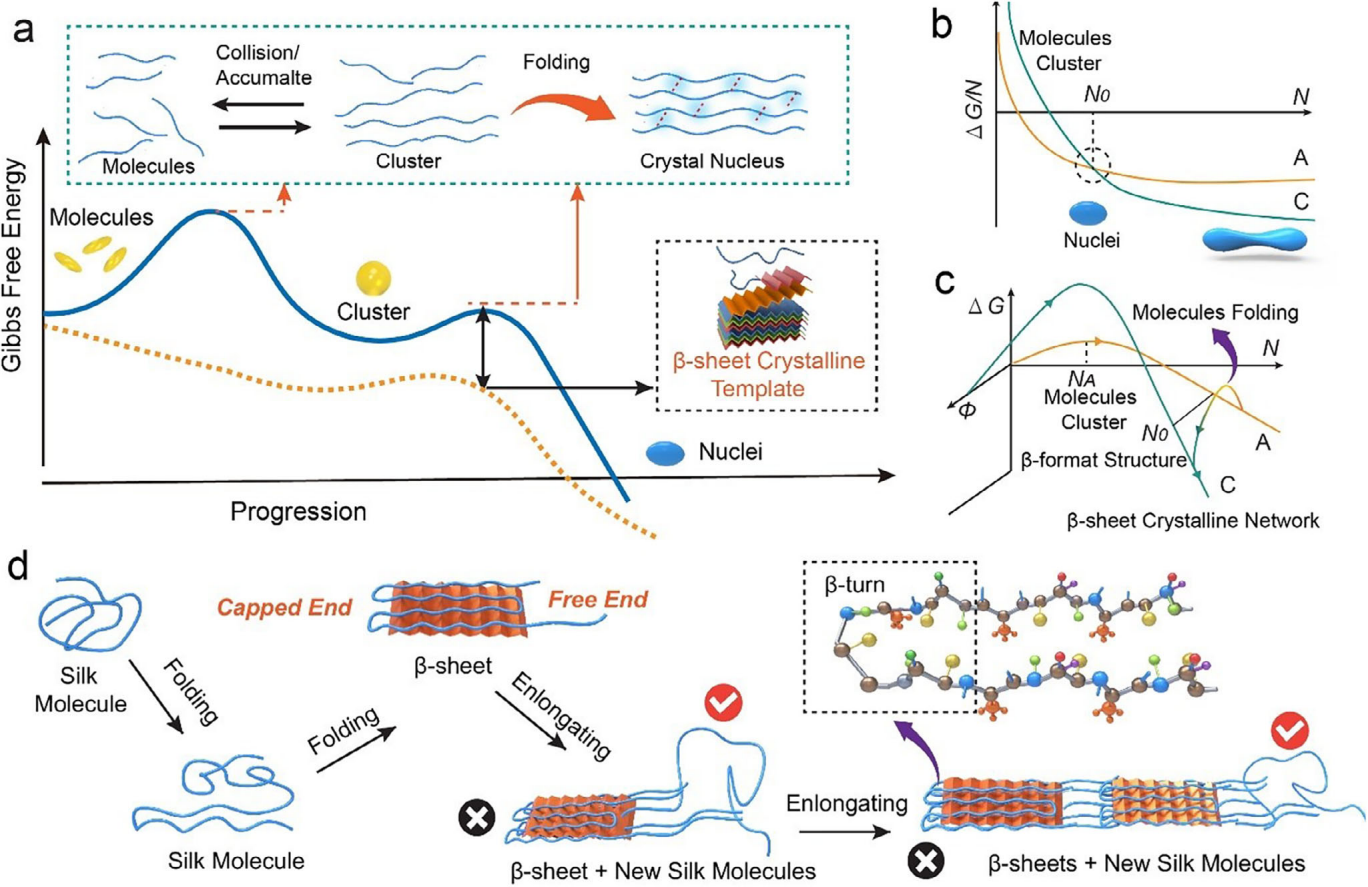
Assembly pathway of silk nanofibril. a) Proposed energy diagram of assembly from molecules to nanofibrils. In overcoming the first energy barrier, unfolded silk molecules aggregate to form a disordered silk cluster. In overcoming the second energy barrier, the aggregated molecules relax into the β‐sheet conformation through intra‐ and inter‐molecular hydrogen bonding. b) Free energy of formation (ΔG) as a function of size exhibits a crossover between crystalline (C) and amorphous (A) phases at N₀. c) The ΔG curves for both crystalline (C) and amorphous (A) forms indicate that the direct barrier is larger for C compared to A. However, transitioning from A to C at a larger size may bypass this barrier. Φ represents the degree of molecular order. d) Proposed directional growth process of silk nanofibrils.

Our findings demonstrate that silk nanofibril assembly follows a two‐step mechanism (**Figure** **7**a) associated with size‐dependent phase stability (Figure 7b). In the first step, individual molecules aggregate into amorphous clusters that are unstable and fluctuate in size until they grow large enough to overcome the free‐energy barrier for forming stable amorphous clusters (Figure 7c).^[^ ^46^ ^]^ In the second step, continued incorporation of silk molecules renders the cluster metastable as it exceeds the size N_0_ at which the crystalline phase becomes more stable.^[^ ^33^ ^]^ At a size above N_0_, the energy barrier to the conformational transition becomes sufficiently small that it is overcome, thus generating stable nuclei of the crystalline phase (Figure 7c).

The establishment of highly ordered hydrogen bonds drives the transformation of disordered, unfolded silk molecules into the more compact β‐sheet silk structure (Figure S13, Supporting Information), thus causing a contraction of the cluster (Figure 7a; Figures S13–S15, Supporting Information).^[^ ^6^, ^47^, ^48^ ^]^ This is shown by the observation that small clusters gradually degrade when treated with papain enzyme, while clusters larger than ≈3.5 nm remain stable and are thus predominantly crystalline (Figure S16, Supporting Information). Thus, the results show that silk follows the two‐step energetic and structural pathway that many other proteins are known to exhibit: the lowest energy pathway to nucleation bypasses the high barrier associated with directly forming the ordered state, instead taking an easier route via the amorphous state.^[^ ^34^ ^]^

Although we do not have molecularly resolved data to observe the transformation process, we can logically expect that diffusion and collision of silk molecules start the process of forming a new cluster. Through inherent thermal fluctuations, the molecules establish contacts between their amino acid residues and realign these contacts to find conformations that minimize the free energy of the emerging structure (Figure 7a). Above the size N_0_ that minimum corresponds to the crystalline phase. Thus, the continual adjustment results in a compact β‐sheet crystalline structure and, thus, a noticeable contraction.

A similar two‐step process occurs repeatedly at the nanofibril tip during the elongation process: silk molecules add to the tip in a disordered state before undergoing conformational transition, which is seen morphologically as a dimensional contraction. Thus, the elongation process can be seen as a repetitive nucleation process in which the newly formed nanofibril segments are generated from a new “nucleation” event at the nanofibril tip. However, the observation of directional growth in the silk nanofibril is noteworthy as it occurs without the need for external energy input. Since the energy required to form a new cluster in either direction is equivalent, the differing growth rates at the two ends must be attributed to kinetic factors. We propose that the β‐turn structure acts as a loop, linking adjacent β‐strands to form a β‐sheet structure (Figure 7d), resulting in one end being capped. Silk molecules are kinetically favored to accumulate in the opposite direction, where they form a cluster, leading to growth at only a single end (Figure 7d). This is similar to the preferential addition of actin subunits at one end of a filament, where they bind stably and enable growth from that end only, while subunit addition at the other end is conformationally unfavorable.^[^ ^35^ ^]^ We additionally find that the propensity for formation of the amorphous clusters is highly dependent on the reaction conditions. For example, the length of nanofibrils is positively correlated with the concentration of silk molecules (Figure S17, Supporting Information), as is the probability of nanofibril branching induced by the development of a cluster on the body of the nanofibril. Therefore, at high concentrations, silk forms a network of long nanofibrils (Figure S18, Supporting Information).

The various internal conformations not only add complexity to the growth process but also influence the final nanofibril morphology. The twisting structure of silk nanofibrils likely arises from the competitive interplay between elasticity and electrostatic repulsion. Like charges on amino acid side groups, positioned closely to each other on the nanofibril, should increase their mutual distance through electrostatic repulsion, but greater distances between consecutive segments of the nanofibril are penalized by elastic energy, therefore favoring a flat structure that is as relaxed as possible. The overall outcome is a twisted morphology. However, unlike amyloid fibrils, which possess homogenously twisted ribbons that arise from orderly stacked β‐sheets perpendicular to the fiber axis,^[^ ^49^ ^]^ the heterogeneous twist observed in silk nanofibrils may result instead from the presence of non‐β‐sheet (i.e., random coil) structures connected by parallel β‐sheets along the fibril axis, which are randomly distributed within the nanofibril.

Finally, the increase in growth rate, length, and resistance to enzymatic digestion achieved by first digesting disordered regions to create β‐sheet seeds demonstrates that the energy landscape for the assembly of silk can be modified by this method to create a lower energy pathway (yellow dashed line in Figure 7a) that proceeds directly to the ordered state, bypassing the disordered cluster state. Modifying the energy landscape necessarily changes the kinetics of assembly and, consequently, influences the ultimate product, allowing for the possibility of directed modification of these materials (Figure 4d). Overall, by employing this reconstruction method, we may ultimately be able to design silk materials with unique physical properties, such as resistance to enzymatic degradation and greater mechanical strength (Figure 6g; Figure S19, Supporting Information). Given the importance of similarly complex biopolymer nanofibrils in many biological materials, these findings provide a potential blueprint for manipulating the structure of these materials for specific technological applications, provided these fundamental nucleation and growth mechanisms are first understood. By modifying the energy landscape that defines these processes, we may be able to precisely direct crystallization, allowing for much greater control over the local and overall crystallinity of the final product.

## 4 Experimental Section

### Material

Silk protein was regenerated from the cocoons of domesticated *B. mori* silkworms (Guangxi Sericulture Technology Co., Ltd.). 20 g of cocoons were boiled twice for 30 mins in an aqueous solution of Sodium bicarbonate (NaHCO_3_) (0.02 m), then thoroughly rinsed with pure water to remove the sericin and NaHCO_3_. The cocoons were dried overnight at 50 °C in an oven. The degummed dry silk fiber (10 g) was dissolved in Lithium bromide (LiBr) solution (9.3 m, 60 mL) at 60 °C for 4 h. The silk protein solution was obtained by extracting LiBr from the dissolved mixture using a dialysis cassette (Solarbio, molecular weight cut‐off 3500) for 2.5 days with frequent changes of deionized (DI) water. The final regenerated silk protein solution was filtered with gauze. The fresh silk protein solution was injected into liquid nitrogen through a syringe pump to form small globules and then freeze‐dried for 3 days. The dried silk globules were stored at −20 °C before further usage. NaHCO_3_ and isopropanol were obtained from Sinopharm Chemical Reagent. LiBr was provided by Aladdin Industrial Corporation.

### In Situ AFM Characterization

Silk protein powders were mixed with 10 mL nuclease‐free water (Ambion, USA), was purified with a 0.2 µm filter to remove aggregate and insoluble protein. 80 µL of silk protein solution was added on top of a freshly cleaved highly oriented pyrolytic graphite (HOPG) surface at room temperature. In situ images were captured using silicon probes (SNL‐C, k: 0.24 N m^−1^, tip radius: 2 nm; Bruker) under tapping mode with a Cypher ES AFM (Asylum Research). The in situ enzymatic perfusion AFM characterization was performed using an AFM fluid cell within the Multimode AFM (Nanoscope V controller, Veeco Metrology, Inc., Santa Barbara, CA, USA). The enzyme solution was perfused into the AFM liquid cell during the silk nanofibril assembly process. The papain (≥10 U mg^−1^ protein) and the protease XIV (≥3.5 U mg^−1^) were purchased from Sigma–Aldrich. The concentration of papain and protease XIV used in the AFM experiment was 125 µg mL^−1^. Images were analyzed by Gwyddion SPM data analysis software. To obtain the width of nanofibrils, tip convolution (dilation) was corrected. When scanning an object with an elliptical cross‐section, the AFM tip artificially broadened the measured width (Figure S20, Supporting Information). For a steep‐walled tip, the true width (W_true_) was related to the measured diameter (W_ex_) by a simple equation: W_true_ = W_ex_‐(h_ex_‐δ_h_‐2r)tanθ‐2r. Where h_ex_ is the experimentally measured height, W_ex_ is the experimentally measured width, W_true_ is the actual width of tested nanofibril, δ_w_ is the added width due to tip convolution, δ_h_ is the eliminated height of the ellipse, which is set to zero in the equation to obtain the upper limit on broadening, r is the tip radius, and θ is the angle made by the tip wall and the normal to the surface. The values of 𝑟 and θ are determined by the tip manufacturer: SNL‐c tip: r = 2 nm, θ = 25°, yielding a calculated W_true_ ranging from 6 to 10 nm.

### Nanomechanical Test

Fast force mapping in liquid was performed using the Contact Fast Force Mapping mode on a Cypher VRS AFM (Asylum Research) with AC55TS probes (Oxford Instruments), featuring a spring constant (k) of ≈85 N m^−1^ and a resonance frequency (f) of 1600 kHz. The typical scanning rate was 0.62 Hz. A deflection setpoint of ≈2 nN was carefully controlled to ensure high‐quality force curves while preserving surface topography. Image processing and analysis were conducted using the Igor Pro software package.

For nanomechanical testing in air, samples were prepared following the same incubation protocol as in liquid imaging experiments. Imaging and mechanical property measurements were performed simultaneously using PeakForce QNM mode on a Multimode 8 AFM (Bruker) under ambient conditions. The same AC55TS probes (Oxford Instruments) were used, with a setpoint maintained at ≈20 nN. Image processing and analysis were carried out using the Gwyddion SPM data analysis software. In all the mechanical tests, deformation depths were kept to ≈0.3 nm, which was below 10% of the sample thickness, to eliminate the contribution from the substrate. Please note that silk is highly sensitive to water. In a hydrated state, silk materials can become significantly softer, with their modulus decreasing by an order of magnitude compared to in air.^[^ ^50^ ^]^)″

### PiFM Characterization

All samples were characterized in the air using a VistaScope PiFM (Molecular Vista Inc.), coupled to a Laser Tune QCL with a wave number resolution of 0.5 cm^−^¹ and a tuning range from 800 to 1800 cm^−^¹. The microscope was operated in dynamic mode with HQ/Cr‐Au probes (MikroMasch). The data was processed using Surface Works software (Molecular Vista Inc.). For silk nanofibril samples, an 80 µg mL^−1^ protein solution was incubated on HOPG for 1 h. The solution was then removed using Kimtech wipes, and the sample was dried in air for 12 h. For silk nanosphere samples, a 90 µg mL^−1^ protein solution was incubated on HOPG for 5 mins and then quickly dried in a 60 °C oven, or a 30 µg mL^−1^ protein solution was incubated on Mica until it naturally dried in air.

### CryoEM Characterization

Cryo TEM was performed on a JEOL GrandARM‐300F, equipped with a Gatan OneView IS camera. A lacey carbon film Cu TEM grid was glow‐discharged for 25 s at 15 mA using PELCO easiGlow before the vitrification. 3 µL of reaction solutions were vitrified using an FEI Vitrobot Mark III, which was blotted for 4 s and plunge‐frozen in liquid ethane. Images were obtained with a defocus value in the range of −1 to −2 µm. The accumulated total dose was less than ≈4 e^−^Å^−2^.

### Attenuated Total Reflection Fourier Transform Infrared (ATR‐FTIR)

The ATR‐FTIR measurements were conducted by an FTIR Perkin Elmer Frontier with a diamond crystal ATR module. The spectra were collected with 4 cm^−1^ resolution in the spectral range from 4000 to 400 cm^−1^ averaging over 64 scans and normalized to the light intensity.

## Conflict of Interest

The authors declare no conflict of interest.

## Supporting information



Supporting Information

## Data Availability

The data that support the findings of this study are available in the supplementary material of this article.
