# Supplementary material for: Manipulating the Assembly and Architecture of Fibrillar Silk
Source: Adv Mater. 2025 Apr 8;37(24):2501096. doi: 10.1002/adma.202501096 (PMC12177862; doi:10.1002/adma.202501096)
Supplement: Supplementary file 1 — Supporting Information [file ADMA-37-2501096-s001.docx]

**(Supporting Information)**

**Manipulating the Assembly and Architecture of Fibrillar Silk**

*Chenyang Shi, Yuna Bae, Mingyi Zhang, James J De Yoreo^*^*

C. Shi, Y. Bae, M. Zhang, J. J. D. Yoreo

Physical Sciences Division, Pacific Northwest National Laboratory, Richland, WA 99354

E-mail addresses: [james.deyoreo@pnnl.gov](mailto:james.deyoreo@pnnl.gov)

J. J. D. Yoreo

Department of Materials Science and Engineering, University of Washington, Seattle, WA 98105


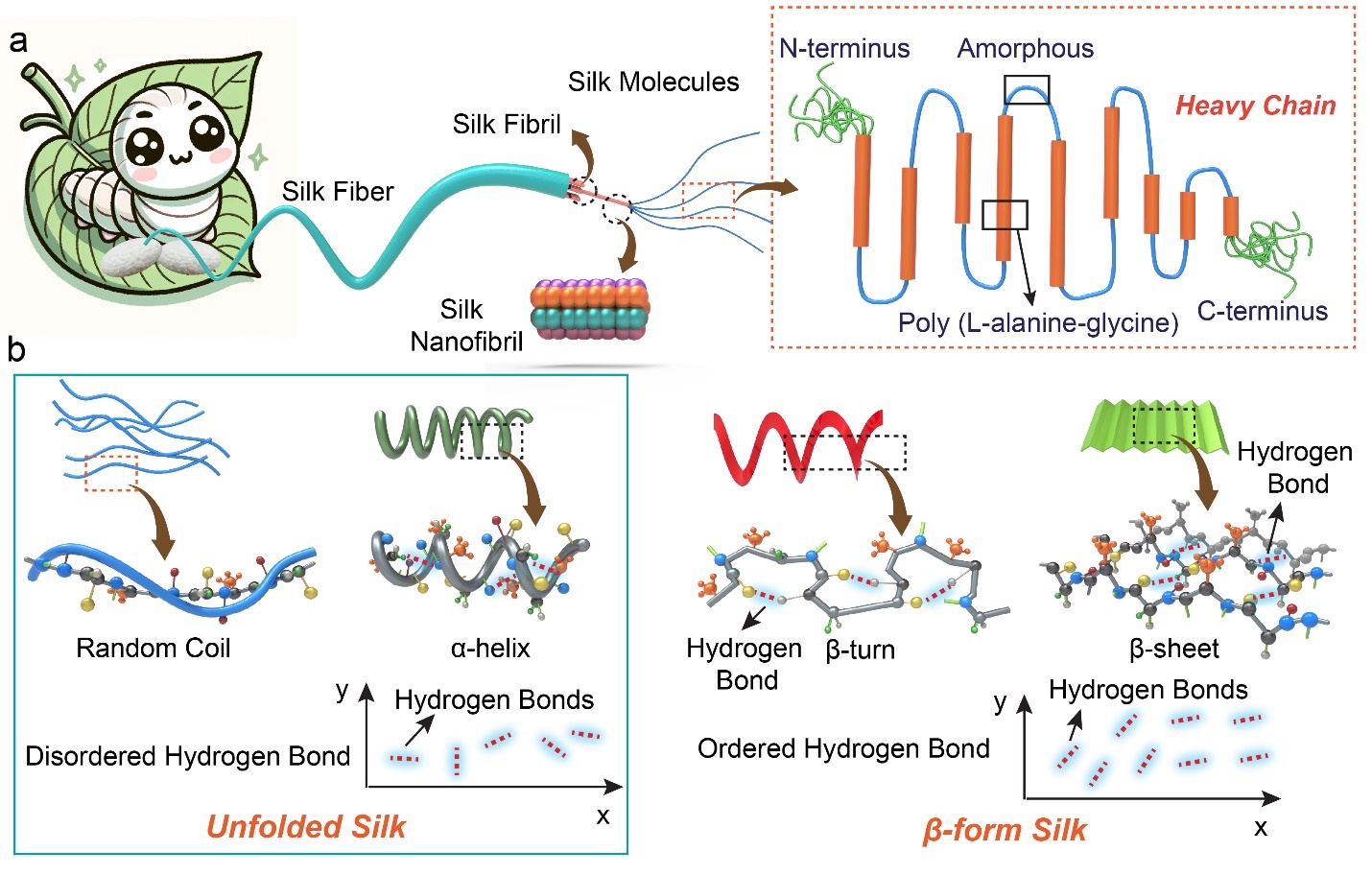


Figure S1. The hierarchical structure in natural silk materials. **a**, the hierarchical arrangement of the Silkworm Cocoon fiber, encompassing various scaled motifs such as fibril, nanofibril, molecule, and heavy chain. **b**, the diverse secondary structures that emerge from silk molecules. The random coil structure and α-helix are regarded as unfolded silk structures, while β-turn and β-sheet are β-form silk structures.

**
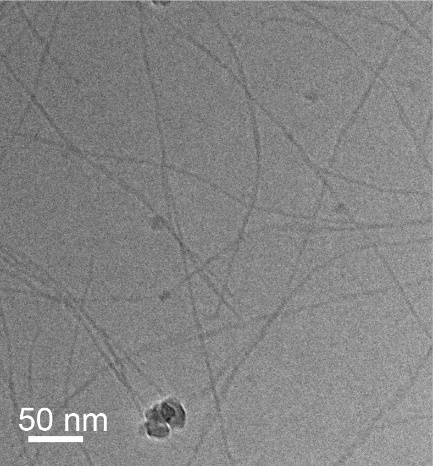
**

Figure S2. The Cryogenic electron microscopy (cryoEM) image of silk nanofibril.


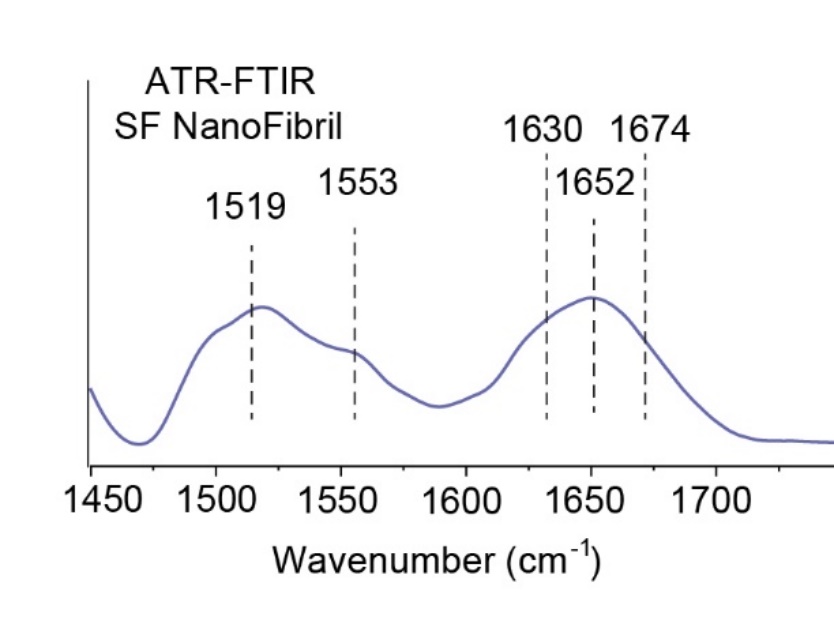


Figure S3. ATR-FTIR spectrum of silk nanofibril. 1630 cm^-1^ represent β-sheets, 1652 cm^-1^ represents unfolded structure, 1674 cm^-1^ represents β-turn.


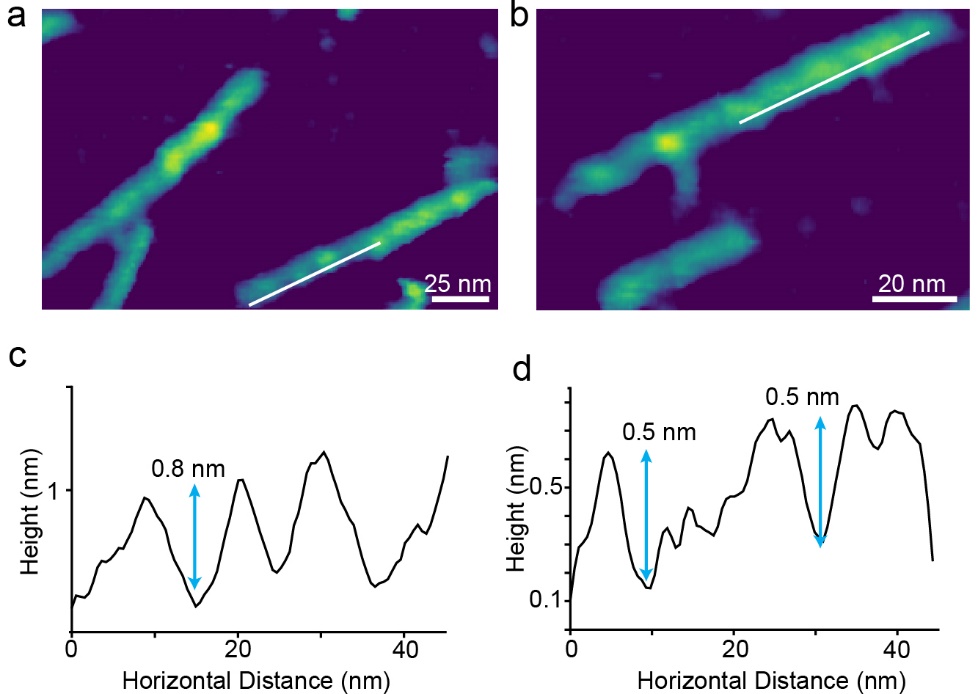


Figure S4. Vertical height difference on the nanofibril. a, b, AFM images of silk nanofibrils. c, d, Related height profiles of nanofibril in image a and b, respectively.


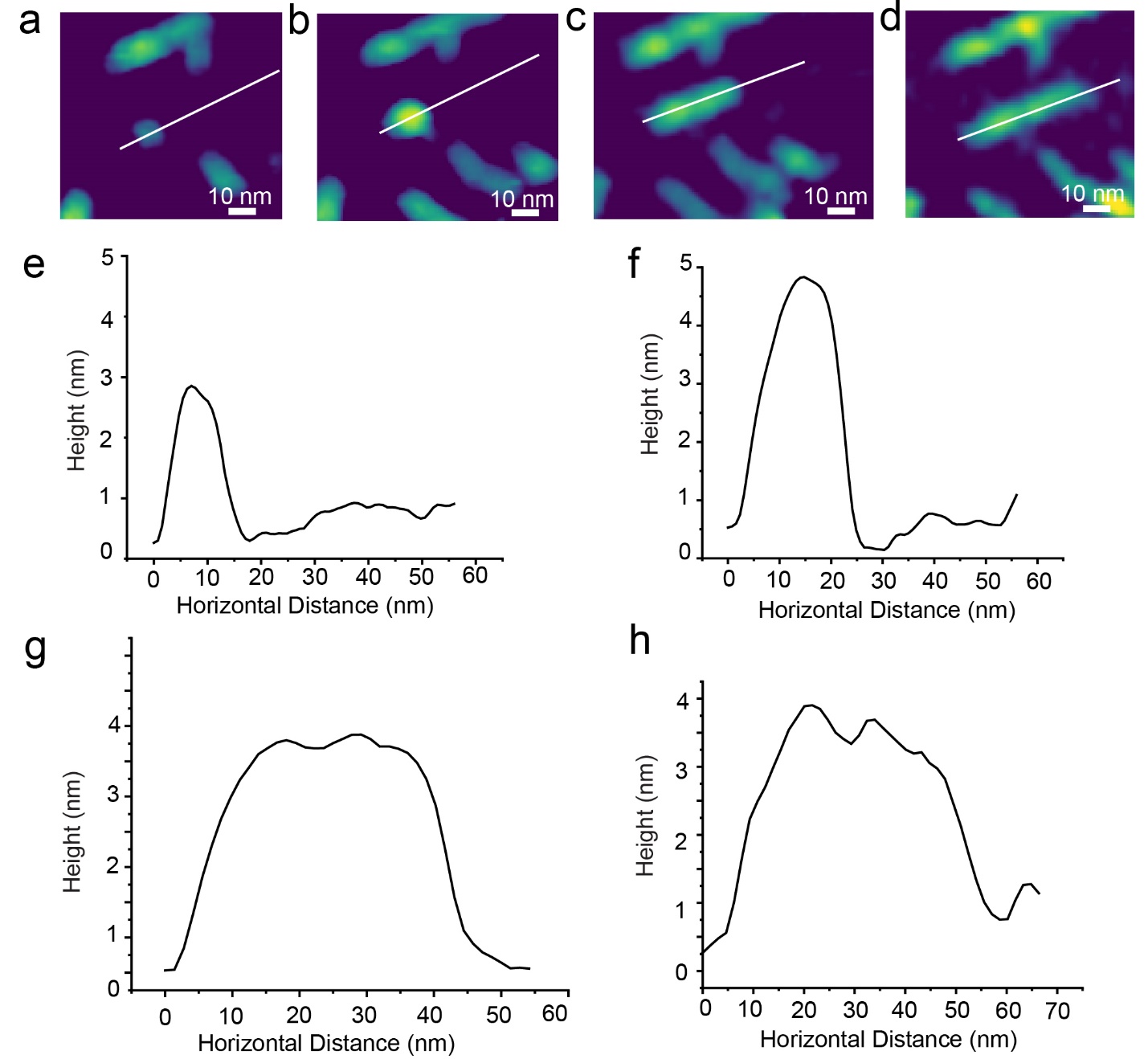


Figure S5. Time-resolved AFM height images (a: 15 min, b: 40 min, c: 60 min, d: 80 min) and corresponding height profile (e-h) of SF nanofibrils showing the elongation process.


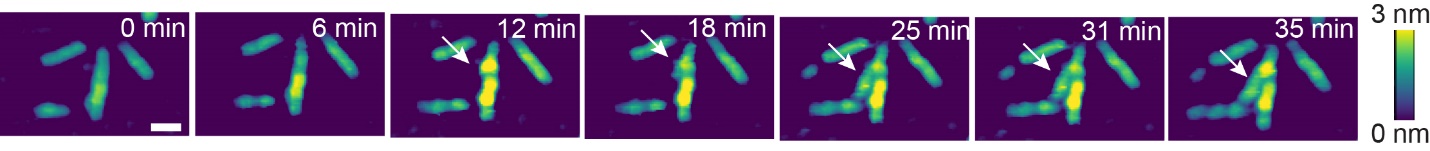


Figure S6. The in-situ time series AFM captures the growth of SF nanofibril branches. Scale bar: 10 nm.


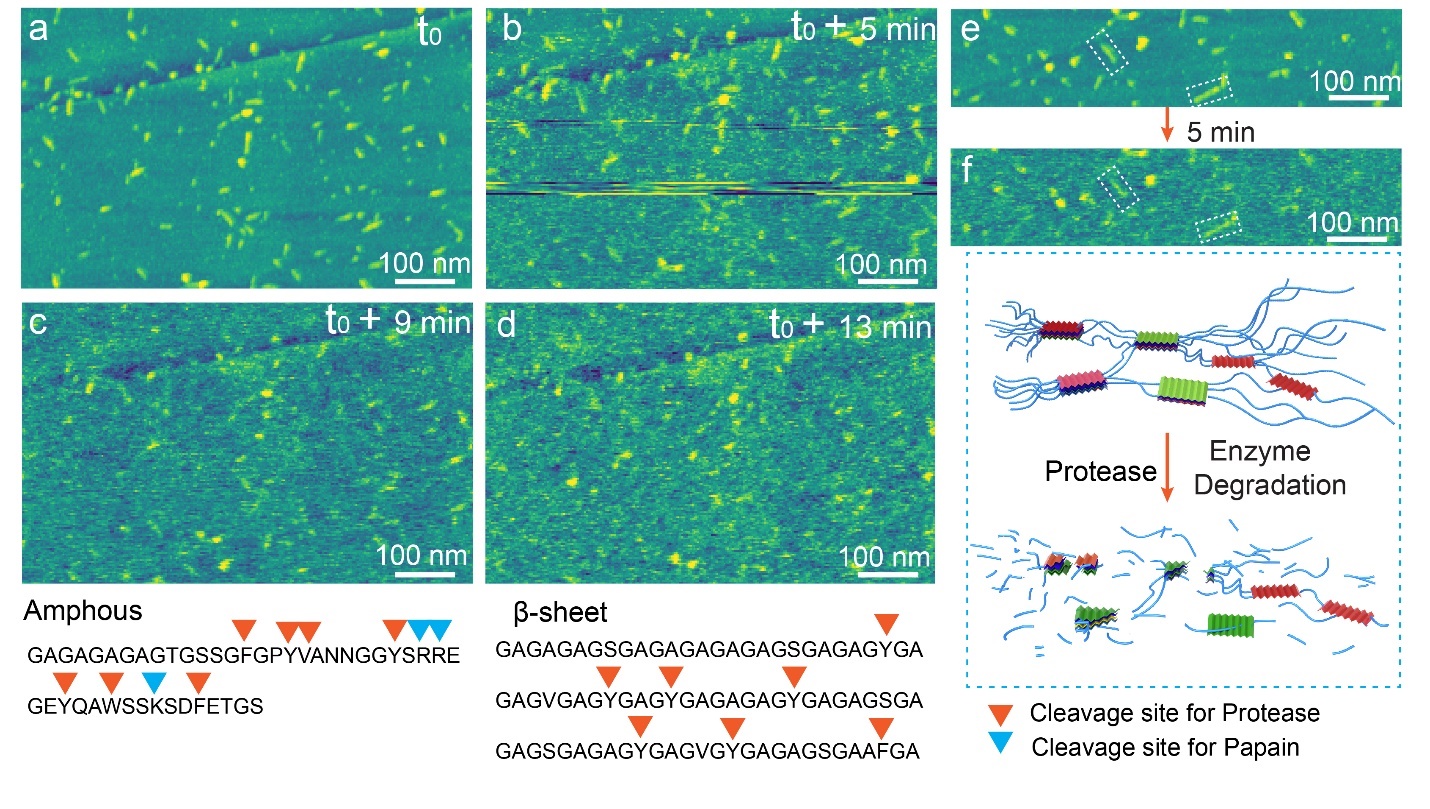


Figure S7. The in situ degradation process of Protease XIV enzyme. Images a-d present time-resolved AFM images illustrating the degradation of silk nanofibrils. Images e-f demonstrate that the nanofibril degradation occurs progressively along the fibril axis, rather than initiating from the tip. This observation suggests that the breakdown of conformations within the nanofibril is non-selective, as shown in the model.


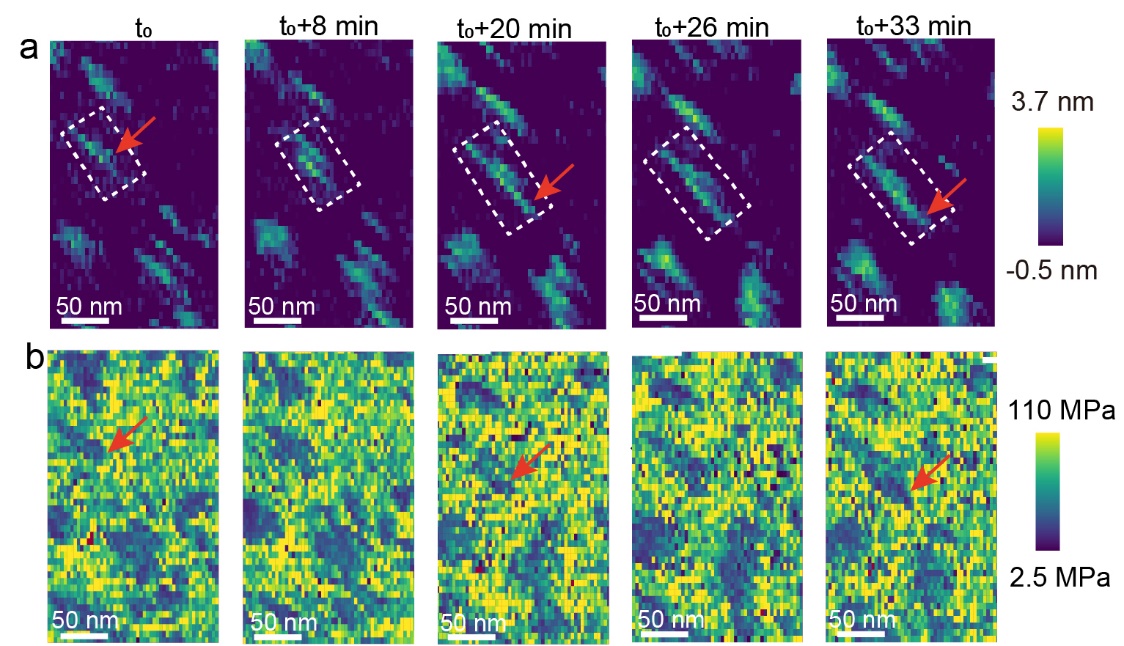


Figure S8. The mechanical measurement of nanofibril by liquid Fast Force Mapping AFM. a) AFM image of nanofibril. b) The corresponding Young's modulus map of nanofibrilThe red arrow points to the tip position of nanofibril, the Young's modulus at the tip is below 10 MPa while the body part is about 30 MPa.


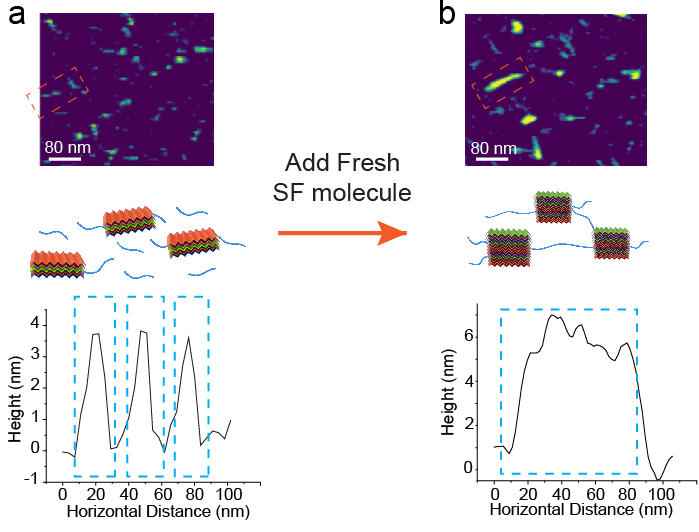


Figure S9. Silk nanofibril reconstruction process. a, from top to bottom: AFM image, scheme, and height profile of β-sheet crystalline. b, from top to bottom: AFM image. scheme and height profile of reconstructed nanofibril.


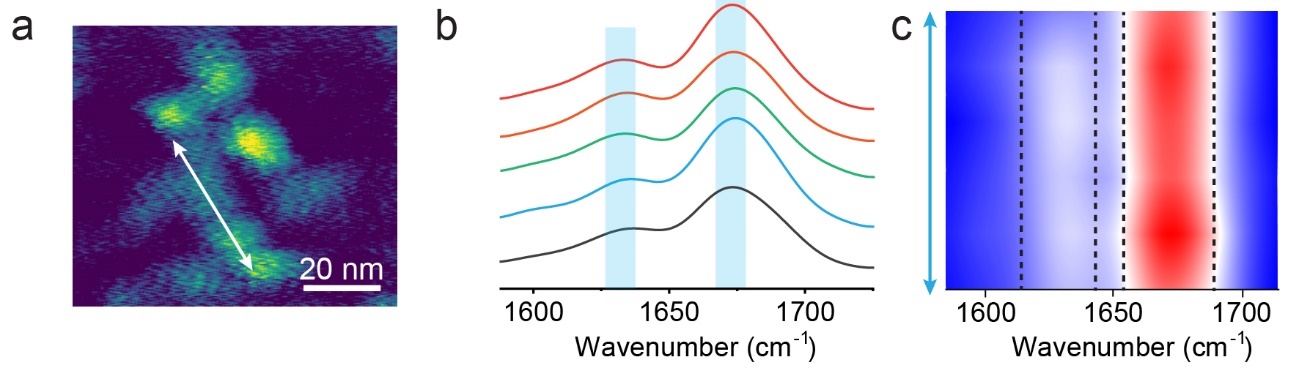


Figure S10. a, AFM image of a nanofibril. b, PiFM spectrum of the nanofibril. c, Color map illustrating the distribution of conformations along the white double arrow line indicated in the image (a).


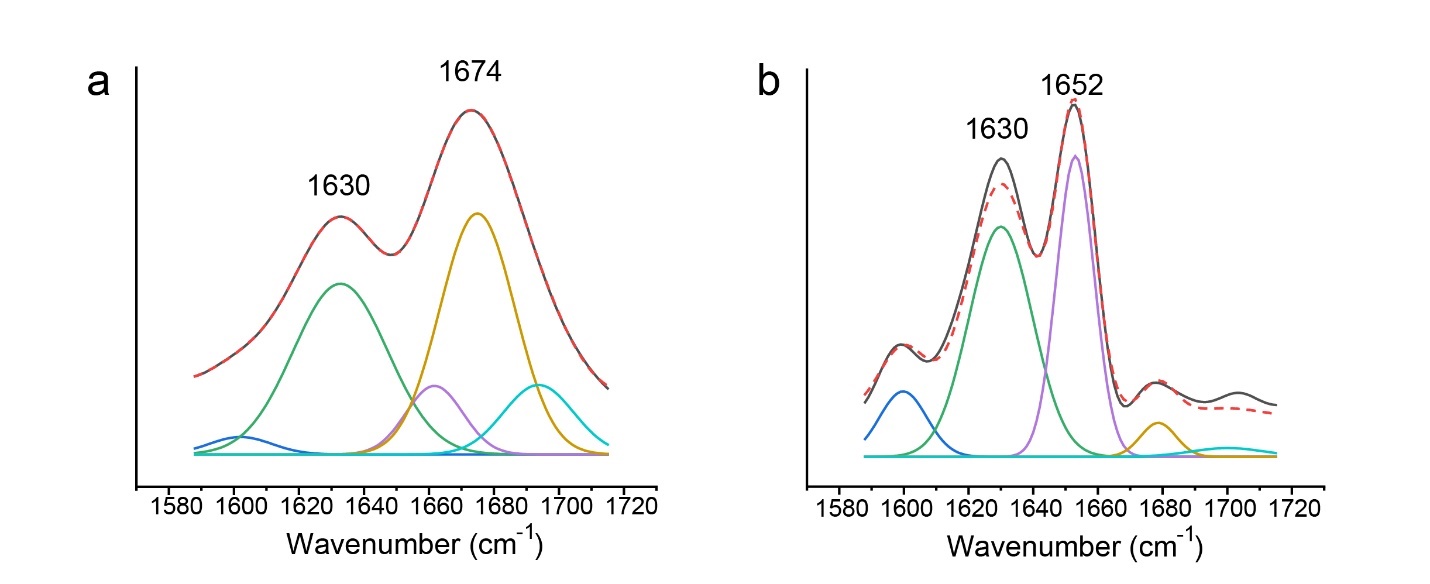


Figure S11. The curve peak fitting of PiFM spectrums. a, the silk nanofibril. b, the silk nanosphere.The small peaks (from left to right) located at 1605 cm^-1^, 1630 cm^-1^, 1652 cm^-1^, 1674 cm^-1^and 1692 cm^-1^, respectively. 1630 cm^-1^ and 1692 cm^-1^ represent β-sheets, 1652 cm^-1^ represents unfolded structure, 1674 cm^-1^ represents β-turn.


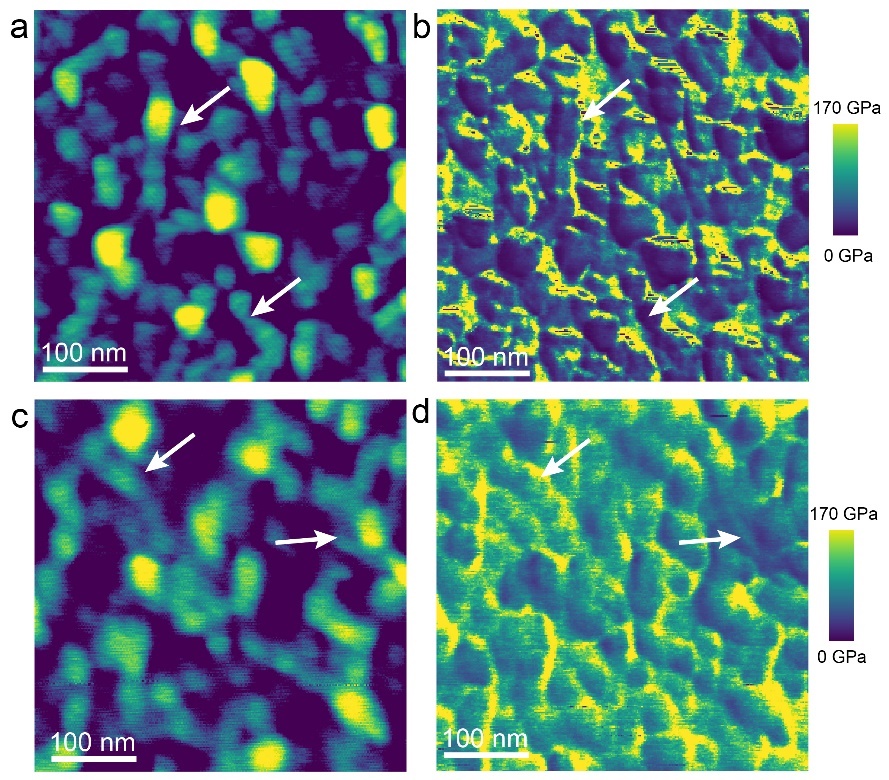


Figure S12. The mechanical measurement of nanofibril. a) AFM image of nanofibril. b) Young’s modulus maps of nanofibril. The Young’s modulus value is 25±5 GPa. c) AFM image of reconstructed nanofibril. b) Young’s modulus maps of reconstructed nanofibril. The Young’s modulus value is 70±10 GPa. The reconstructed nanofibril possesses a bigger size than regular nanofibril. The white arrows point to nanofibril.


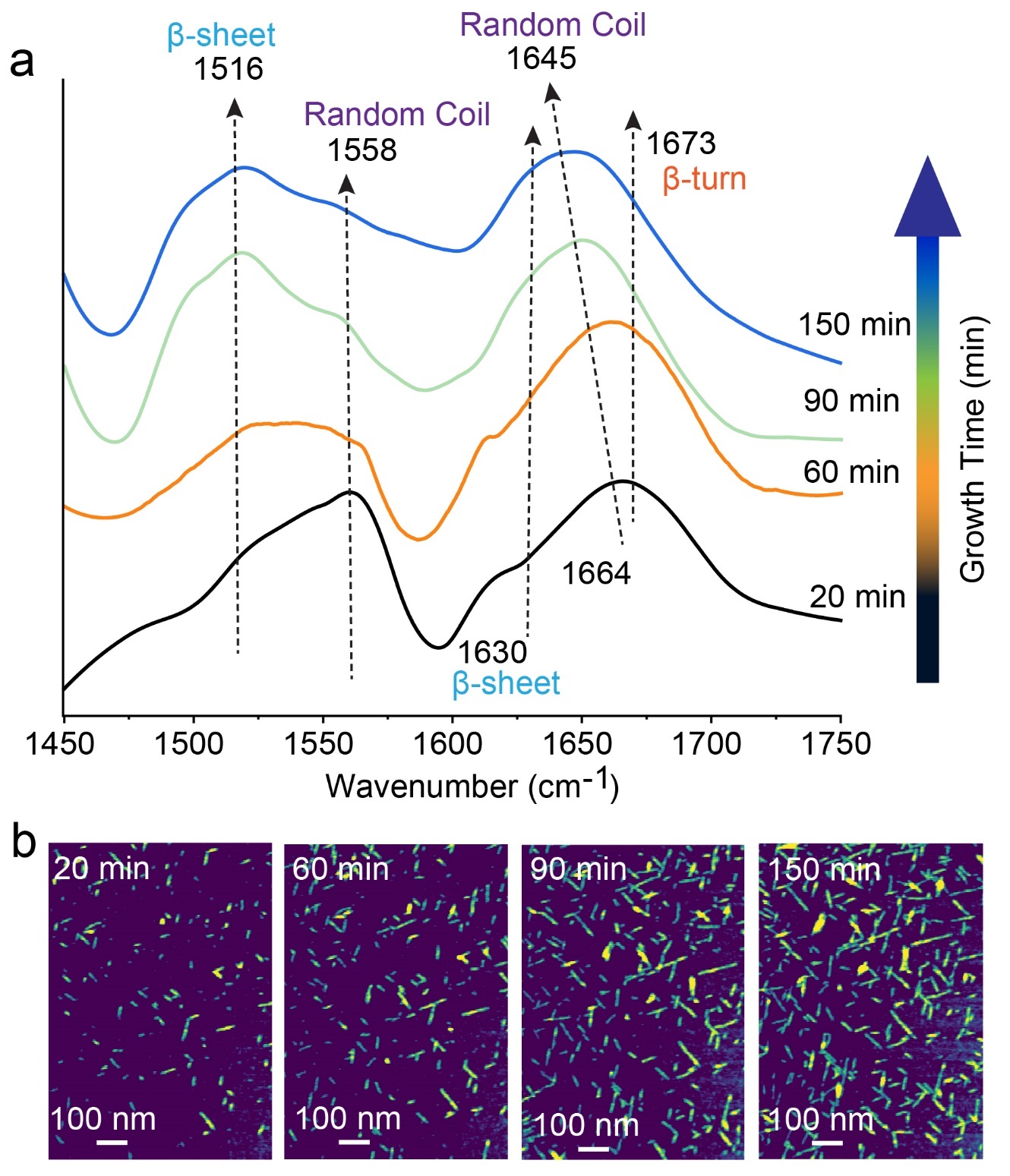


Figure S13. a, ATR-FTIR spectrum of silk nanofibrils with different growth times. b, The corresponding AFM images. We can observe the transformation of unfolded silk molecules (random coil) into β-sheets, accompanied by the elongation of the silk nanofibril.


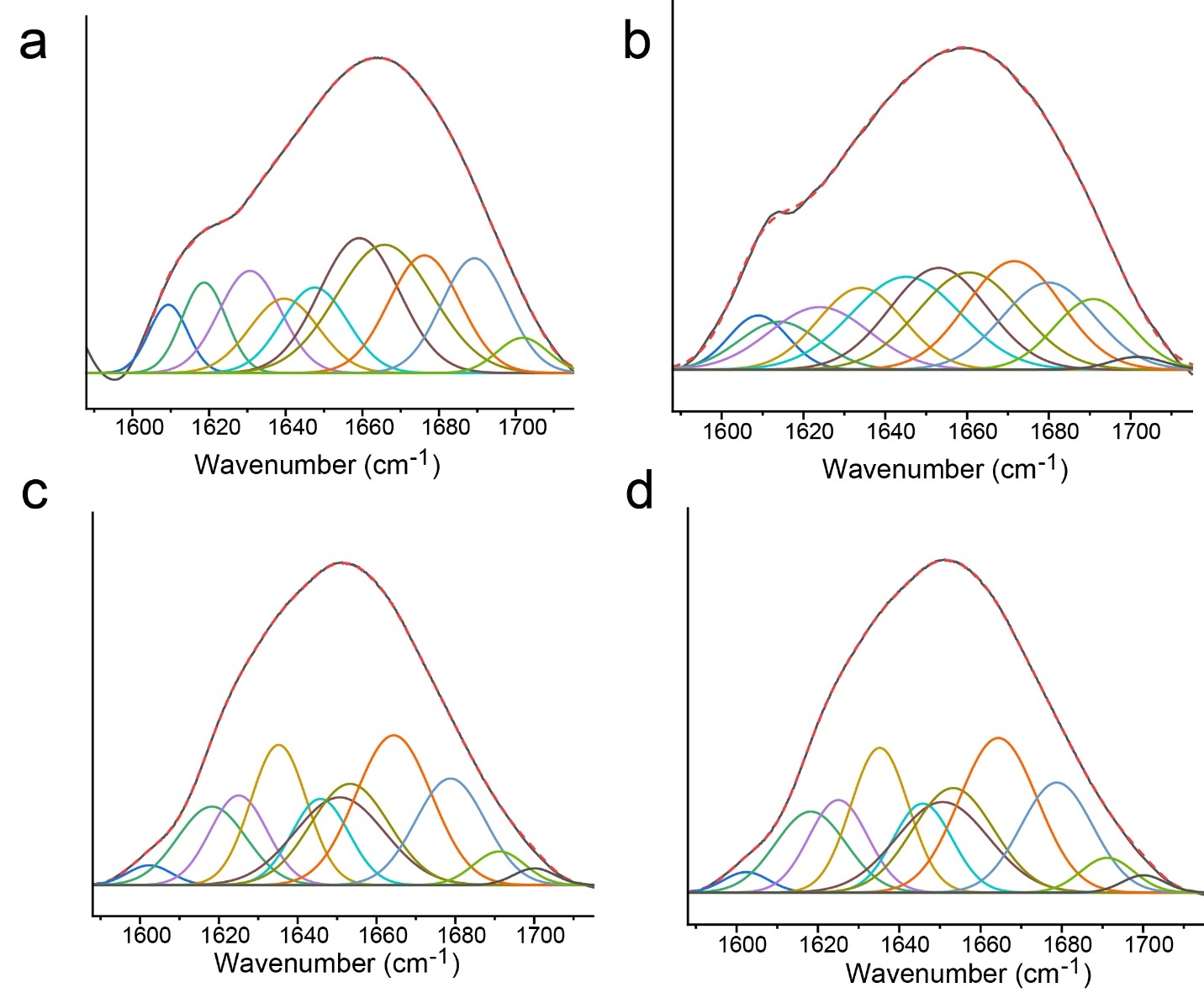


Figure S14. The curve peak fitting of ATR-FTIR spectrums. a, growth time of 20 min, b, growth time of 60 min, c, growth time of 90 min, d, growth time of 150 min.


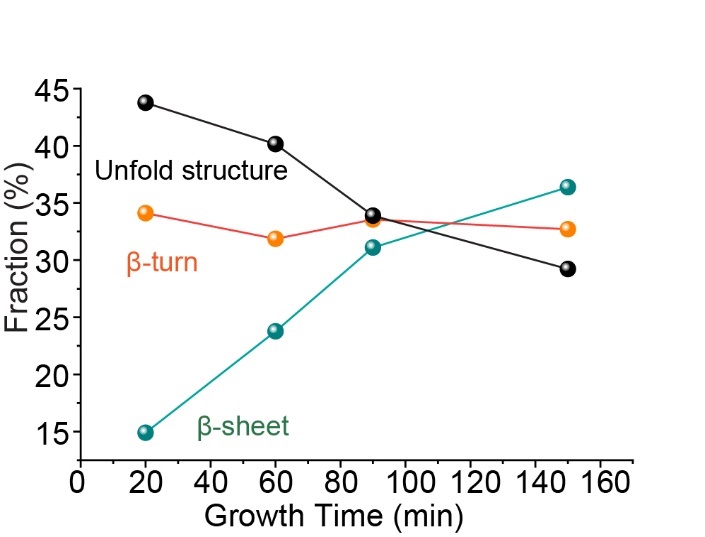


Figure S15. The fraction of various secondary structures in different growth times. The majority of the unfolded structures convert into β-sheets during the assembly of silk nanofibrils.

**
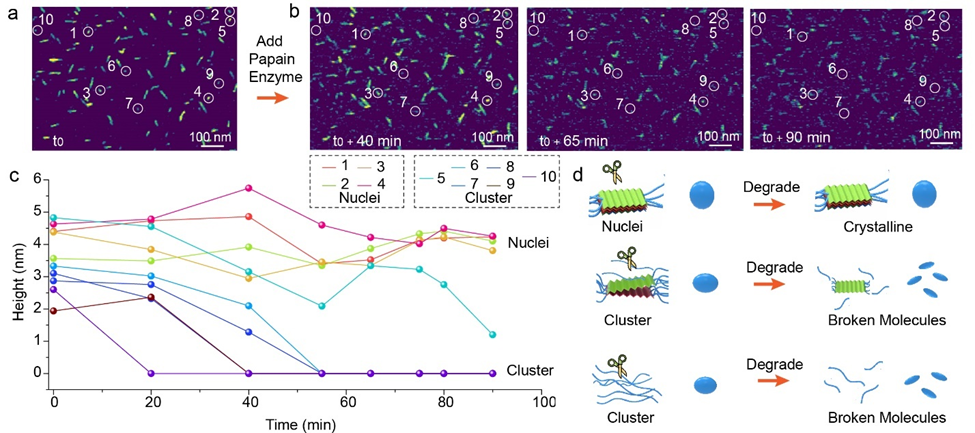
**

Figure S16. AFM height images of silk nanofibrils (a), and after adding papain enzyme (b). c, The corresponding height profile of objects in images a and b. Numbers 1-4 maintain their size without degrading, while numbers 5-10 gradually degrade with enzyme treatment. d, The schematic shows the different phase states of silk clusters and nuclei.


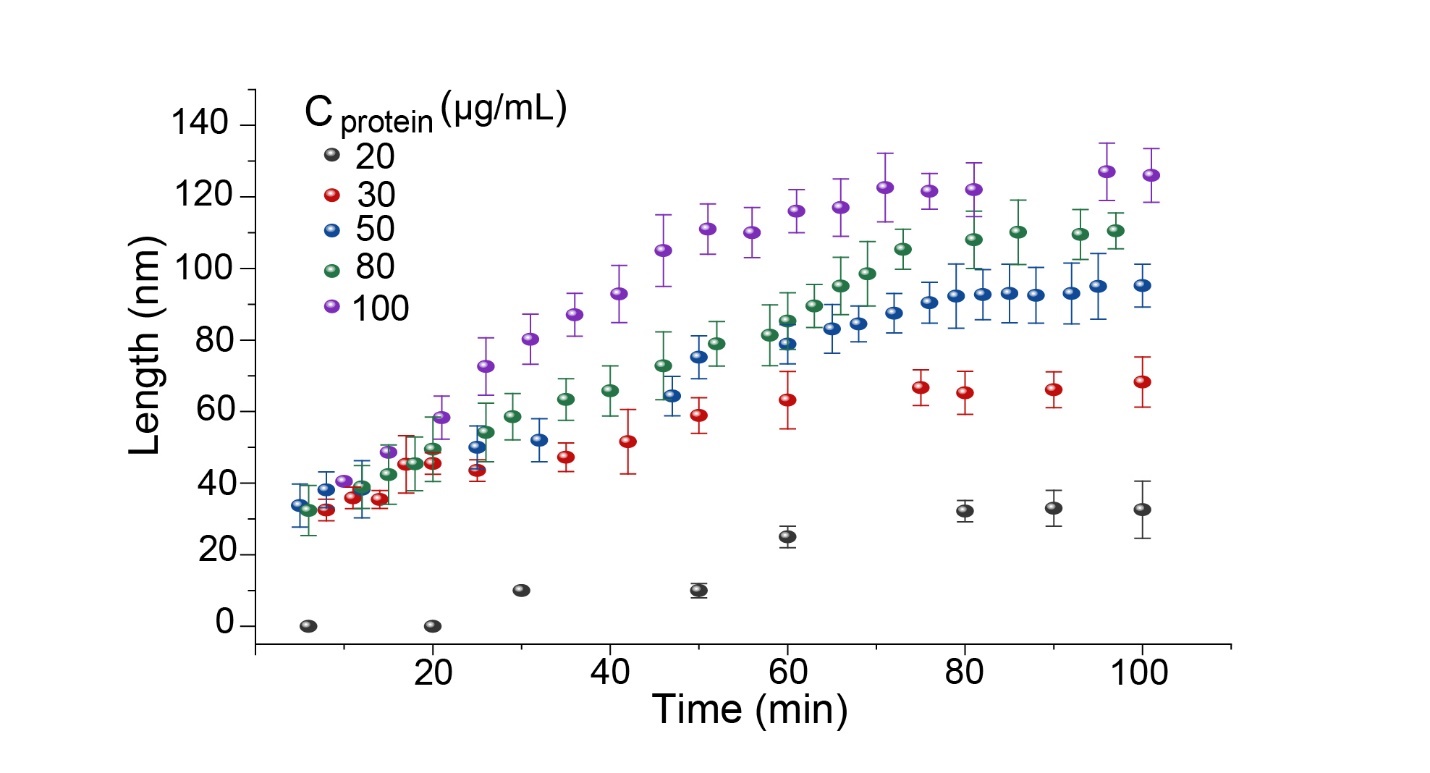


Figure S17. Variations in nanofibrils growth length versus silk protein concentration.


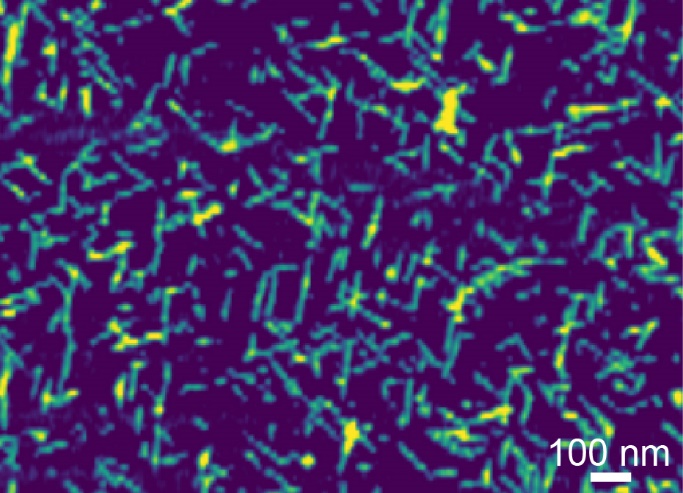


Figure S18. The AFM image of silk nanofibril network.


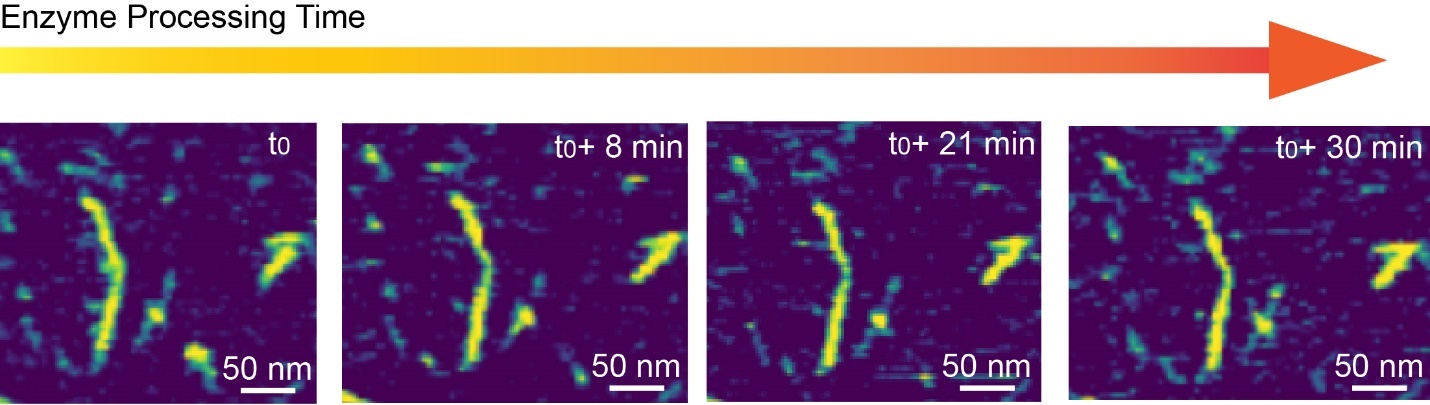


Figure S19. The time-resolved AFM images demonstrate the stability of reconstructed nanofibrils following the injection of fresh enzymes. The shape and length of the reconstructed nanofibril remain unchanged even after prolonged treatment with the enzyme.


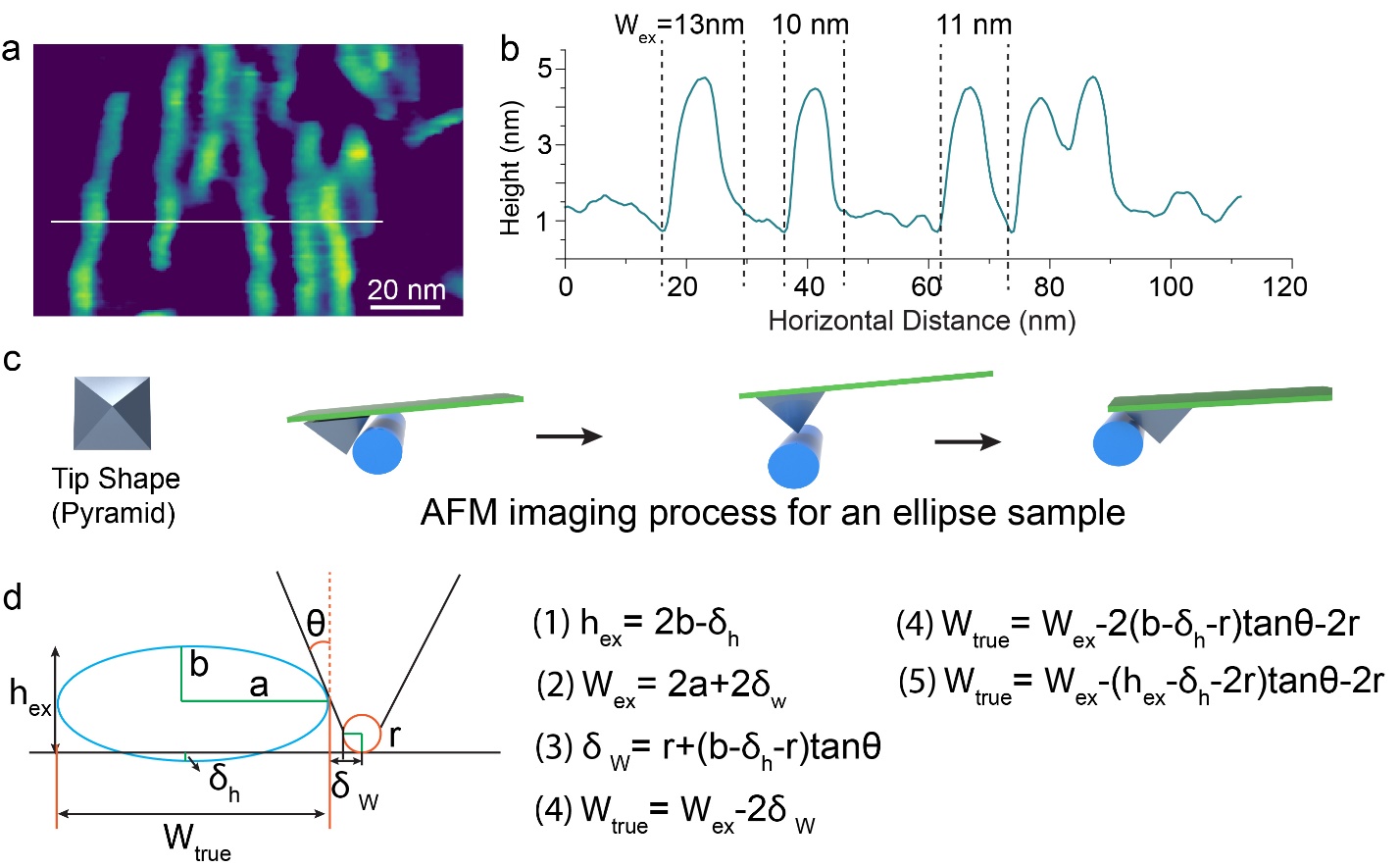


Figure S20. Measurement of a nanofibril. a, AFM image of a nanofibril. b, Corresponding height profile along the white line in (a). c, Schematic illustration of the AFM imaging process for an ellipse using a pyramidal tip. d, Schematic of the deconvolution process, where h_ex_ is the experimentally measured height, W_ex_ is the experimentally measured width, Wtrue is the actual width of tested nanofibril, δ_w_ is the extra broadening, δ_h_ is the eliminated height of the ellipse, which is set to zero in our equation, r is the tip radius, θ is the effective tip angle. The values of 𝑟 and θ are determined by the tip manufacturer: SNL-C tip: r=2 nm, θ=25^o^.
